# Hierarchical Flexible Ceramic Nanofiber Membrane for Simultaneous Membrane Distillation and Photocatalytic Hydrogen Evolution

**DOI:** 10.1002/adma.74777

**Published:** 2026-08-24

**Authors:** Pengfei Lin, Xiaochen Lu, Jiawei Sun, Shuai Guo, Xinping He, Chunhai Yi, Zhenyao Wang, Jiaxin Guo, Swee Ching Tan, Alicia Kyoungjin An

**Affiliations:** ^1^ State Key Laboratory of Fluorine & Nitrogen Chemicals School of Chemical Engineering and Technology Xi'an Jiaotong University Xi'an China; ^2^ Laoshan Laboratory Qingdao China; ^3^ Department of Chemical and Biological Engineering the Hong Kong University of Science and Technology Hong Kong China; ^4^ Department of Materials Science and Engineering National University of Singapore Singapore Singapore; ^5^ Department of Environmental Science and Engineering Macau University of Science and Technology Macau China

**Keywords:** desalination, hydrogen production, mass transfer, materials science, membrane, membrane distillation, multiphysics, nanotechnology, photocatalysis, solar energy

## Abstract

The escalating demand for clean energy and freshwater necessitates integrated rather than compartmentalized technological solutions. However, conventional desalination and photocatalytic hydrogen production typically operate as disparate processes, resulting in suboptimal solar energy utilization and increased system complexity. Herein, a hierarchical and flexible ceramic nanofiber membrane is engineered to fundamentally couple photocatalytic hydrogen evolution with thermally assisted membrane distillation, enabling the simultaneous generation of freshwater and hydrogen. The La‐doped ZrO_2_ nanofiber scaffold imparts exceptional mechanical robustness and irradiation stability, while in situ‐constructed superhydrophobic UiO‐66‐NH_2_/CdS heterojunctions facilitate highly efficient light harvesting, directed charge separation, and durable anti‐wetting performance. Consequently, the integrated membrane concurrently delivers an outstanding hydrogen evolution rate of 74.8 mmol m^−2^ h^−1^ and a sustainable vapor flux of 41.0 L m^−2^ h^−1^ with 99.95% salt rejection under simulated solar irradiation. Photoelectrochemical measurements and density functional theory calculations elucidate that the configured type‐II heterojunction intrinsically drives spatial charge separation to boost proton reduction. Furthermore, multiphysics simulations demonstrate that the three‐dimensional fibrous architecture remarkably amplifies light confinement and accelerates rapid gas‐product transport, fundamentally mitigating mass transfer limitations. By synergizing structural resilience with unified processes, this work establishes a scalable materials paradigm for high‐efficiency water–energy co‐generation.

## Graphical abstract

A hierarchical flexible ceramic nanofiber membrane integrates a La‐stabilized ZrO_2_ scaffold with superhydrophobic UiO‐66‐NH_2_/CdS heterojunctions. The gas–liquid–solid interfacial architecture sustains vapor‐selective desalination, visible‐light charge separation, and rapid H_2_ extraction, enabling simultaneous freshwater production and photocatalytic hydrogen evolution while suppressing salt crossover and catalyst flooding.

## 1 Introduction

Against the backdrop of escalating global water scarcity and energy crises, achieving the synergistic, efficient, and sustainable production of freshwater and clean energy has emerged as a critical challenge in environmental and energy research. The United Nations Sustainable Development Goals explicitly emphasize the need for innovative approaches to ensure global access to clean water, while promoting the widespread adoption of renewable energy [1, 2, 3, 4, 5, 6]. Solar‐driven photocatalytic water splitting offers a direct route to green hydrogen [7, 8, 9, 10, 11, 12], while membrane distillation (MD)‐ particularly vacuum membrane distillation (VMD) and other thermally assisted variants (TMD)‐ provides a thermally driven, low‐grade heat‐compatible pathway for desalination. When properly coupled, these processes promise synergistic use of the solar spectrum and operating heat, enabling simultaneous generation of hydrogen and freshwater from saline or contaminated waters with minimal external energy input.

Realizing this promise, however, demands materials and architectures that reconcile competing functional requirements. Photocatalysts must present large accessible surface areas, intimate light–matter interaction, and efficient charge separation, yet conventional powdered catalysts suffer from light scattering, aggregation, difficult recovery, and poor mechanical stability when deployed in flow systems [13, 14, 15, 16]. MD membranes, by contrast, require sustained hydrophobicity, low thermal conductivity, and robust mechanical integrity; many polymeric membranes meet these needs under mild conditions, but degrade under prolonged solar irradiation and elevated temperatures [17, 18, 19], leading to pore wetting, loss of selectivity, and shortened lifetimes. Directly assembling photocatalysts onto polymeric substrates often results in poor interfacial adhesion and rapid catalyst detachment, particularly under harsh desalination conditions. These intrinsic mismatches between catalytic and separation requirements have hindered the practical development of multifunctional water–energy conversion membranes.

Two material strategies have shown particular promise for addressing these gaps. First, metal–organic frameworks (MOFs), such as UiO‐66‐NH_2_ provide tunable porosity, abundant functional sites for nanoparticle anchoring, and favorable electronic structures for heterojunction formation; when combined with narrow‐band semiconductors (e.g., CdS) they can form type‐II interfaces that broaden light absorption and markedly improve photogenerated charge separation and pollutant mineralization [20, 21, 22, 23]. Second, replacing organic polymer supports with inorganic ceramic nanofiber substrates‐ engineered for flexibility and thermal stability‐ can overcome the intrinsic thermal‐and photo‐degradation of polymers while retaining high porosity and low mass‐transfer resistance. Critically, seed‐assisted, in situ growth of MOF/catalyst architectures on such ceramic fibers can produce strongly bound, hierarchical coatings that resist detachment during operation and, after appropriate low‐surface‐energy modification, maintain the anti‐wetting behavior required for MD.

Building on these insights, this study reports a hierarchical flexible ceramic nanofiber membrane system (ZLUC NFMs) that integrates desalination and photocatalytic H_2_ evolution through rational materials design and multiscale interfacial engineering (Figure 1). The key novelty of this work lies in the concurrent integration of three functions that are usually addressed separately. First, a La‐stabilized ZrO_2_ nanofiber scaffold is employed as a free‐standing, mechanically flexible, thermally stable, and irradiation‐tolerant ceramic membrane support, overcoming the brittleness of conventional ceramic fibers and the photo/thermal instability of polymeric MD substrates. Second, a seed‐assisted in situ growth strategy is developed to construct a strongly anchored superhydrophobic UiO‐66‐NH_2_/CdS (UC) heterojunction on the ceramic fibers, allowing the same interfacial layer to function simultaneously as an anti‐wetting membrane distillation surface and as a visible‐light‐active charge‐separation platform for photocatalytic H_2_ evolution. Third, the three‐dimensional fibrous network transforms the conventional liquid–solid photocatalytic interface into a gas–liquid–solid triphase reaction pathway while preserving efficient vapor transport for VMD, thereby coupling light harvesting, charge separation, H_2_ bubble release, and desalination within a single membrane architecture. This integrated structural and functional coupling distinguishes ZLUC NFMs from conventional catalyst slurries, planar catalyst‐coated membranes, and passive hydrophobic MD membranes, and establishes a versatile platform for simultaneous freshwater and hydrogen generation.

**FIGURE 1:**
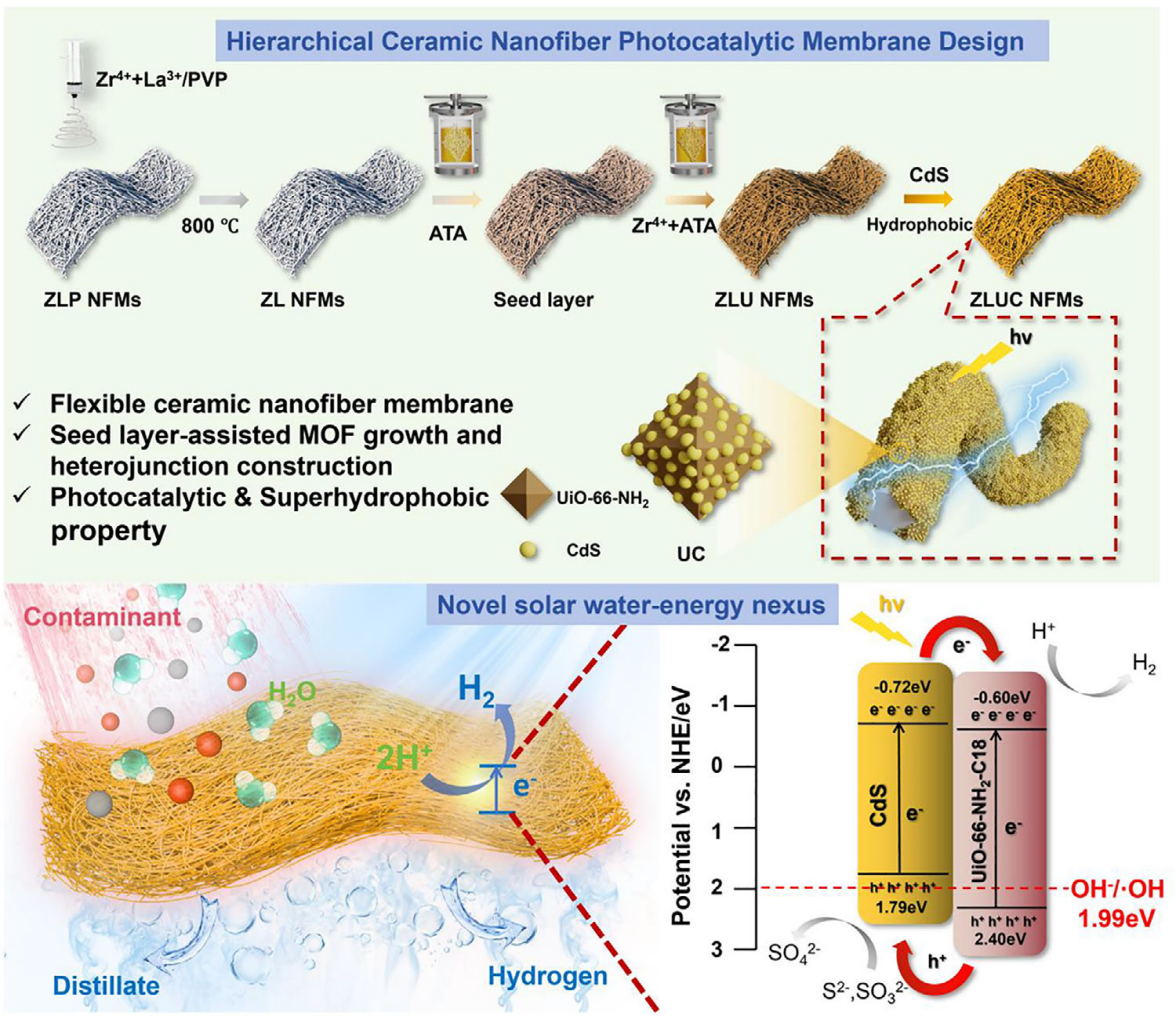
Schematic diagram of the membrane design process and anti‐fouling desalination process for hierarchical ceramic nanofiber photocatalytic membranes.

The synthesis procedure of hierarchical ceramic photocatalytic ZLUC NFMs is illustrated in Figure 1, including the preparation of the spinning solution containing La precursor, electrospinning, calcination to form stable ZL NFMs, UiO‐66‐NH_2_ seed‐assisted growth, in situ growth of UiO‐66‐NH_2_, and the growth of CdS during the hot solvent process followed by hydrophobic modification. From a fabrication perspective, this route has scale‐up potential because the ceramic scaffold is formed by electrospinning/calcination, while the seed‐layer formation, UiO‐66‐NH_2_ growth, CdS deposition, and hydrophobic modification are solution‐based processes performed directly on free‐standing membrane sheets. Larger‐area fabrication could be pursued using continuous or multi‐jet electrospinning and scale‐matched immersion/flow‐through growth reactors, although coating uniformity, solvent recovery, batch reproducibility, and Cd‐containing waste management must be addressed before practical deployment.

## 2 Results and Discussion

### 2.1 Design and Fabrication of Flexible Ceramic NFMs

Conventional ZrO_2_ nanofibers often undergo brittle fracture due to the monoclinic phase transition and grain coarsening during high‐temperature calcination, which severely restricts their structural integrity and practical applicability. Guided by the biphasic morphology of ceramic nanofibers [24, 25, 26] and ZrO_2_ toughening principles [27], a novel microstructural design was engineered for the ceramic nanofibers. The NFMs doped with 0.5 g and 1.5 g of La(NO_3_)_3_ before calcination were denoted as 0.5ZLP and 1.5ZLP, while those after calcination were denoted as 0.5ZL and 1.5ZL, respectively. The thermal decomposition behavior of the 0.5ZLP NFMs was investigated to clarify the removal of the organic spinning components during ZrO_2_ fiber formation. During calcination in air, PVP and acetate species decomposed and could generate transient carbonaceous residues below 500°C, as indicated by the major weight loss and DTA exothermic peaks. These residues were further oxidized and removed during heating from 500 to 800°C, together with dehydroxylation of the ceramic precursor. The disappearance of PVP‐related FTIR bands after calcination further confirmed the removal of polymer‐derived organic residues. Therefore, the final ZL NFMs consist of La‐doped ZrO_2_ ceramic fibers without an intentionally retained carbon phase. Detailed thermal analysis data are provided in Figures S1 and S2.

The surface and cross‐sectional morphologies of pure ZrO_2_, 0.5ZL and 1.5ZL NFMs were examined using a scanning electron microscope (SEM) after calcination at various temperatures, as shown in Figures 2c, d and S3. The 0.5ZLP NFMs exhibited a typical electrospun fiber network morphology at room temperature, with a smooth surface and a fiber diameter of approximately 560 nm. After calcination at 800°C, the fiber curvature of 0.5ZL increased, the surface remained relatively smooth, but the diameter decreased to about 220 nm, which was attributed to the decomposition of PVP (Figure S4). The pure ZrO_2_ fibers calcined at 800°C had a relatively flat and smooth surface, while at 1000°C, the surface became uneven and grainy, indicating that the grain size increased rapidly at this temperature. Both 0.5ZL and 1.5ZL NFMs exhibited smooth and flat fiber surfaces at 800° and 1000°C, suggesting that doping with La(NO_3_)_3_ helped refine the grain size. Notably, some fibers in the 1.5ZL NFMs displayed a banded structure, which was attributed to the excess La(NO_3_)_3_ increasing the conductivity of the spinning solution. The amplitude of the electric field caused by the conductivity affected the formation of uniquely shaped fibers, possibly accelerating the evaporation of solvent molecules and leading to inhomogeneous fiber formation of the polymer solution [28]. The cross‐sectional images of the fibers also showed that the uncalcined 0.5ZLP fibers had a smooth cross‐section without grains (Figure S5), while the grains in the calcined pure ZrO_2_ NFMs were noticeably larger than those in 0.5ZL and 1.5ZL NFMs, further supporting the grain‐refining effect of La(NO_3_)_3_.

**FIGURE 2:**
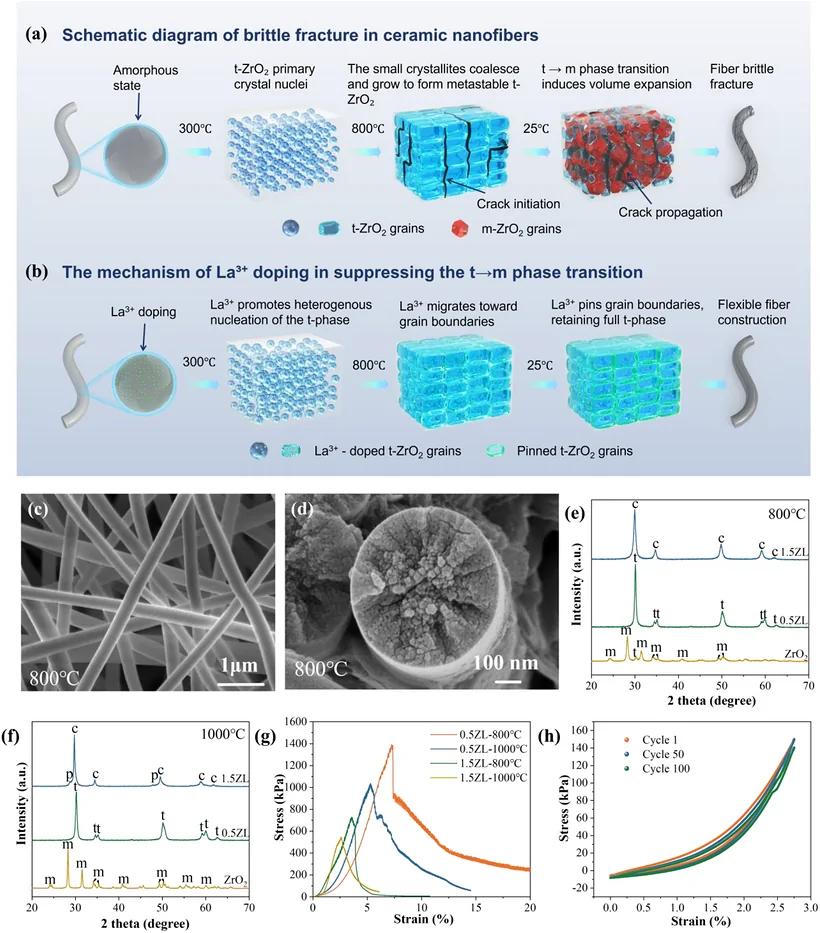
Fabrication and structural characterization of flexible ZrO_2_ fibers. (a) For pure ZrO_2_ nanofibers heated to 800°C and cooled to room temperature, amorphous precursors initially form metastable tetragonal phase (t‐ZrO_2_) nuclei. Coalescence and growth of these crystallites trigger t→monoclinic phase (m‐ZrO_2_) transition with volumetric expansion, leading to fiber brittle fracture. (b) With optimal La^3+^ doping, La^3+^ promotes heterogeneous nucleation of t‐phase, migrates to grain boundaries, and pins them, effectively suppressing t→m transition. This stabilizes the pure t‐phase structure, enabling flexible fiber fabrication. The (c) surface and (d) cross‐sectional SEM images of 0.5ZL formed after calcination at 800°C. XRD patterns of ZrO_2_, 0.5ZL, and 1.5ZL NFMs after calcination at (e) 800°C and (f) 1000°C. (g) Stress‐strain curves of 0.5ZL and 1.5ZL NFMs after calcination at 800° and 1000°C. (h) Fatigue performance testing of 0.5ZL NFMs after calcination at 800°C.

The structure and composition of the samples were analyzed by x‐ray diffraction (XRD). As shown in Figure S6, 0.5ZLP NFMs exhibited no distinct diffraction peaks, confirming their amorphous nature. Upon calcination at 800°C undoped ZrO_2_ predominantly formed m‐ZrO_2_ with minor t‐ZrO_2_ reflections (Figure 2e). The average crystallite size of ZrO_2_ NFMs, determined through the Scherrer equation analysis, was measured to be 18.2 nm. When the temperature was increased to 1000°C, a complete transformation to m‐ZrO_2_ occurred, with crystallites growing to approximately 36.1 nm (Figure 2f). Since this dimension exceeded the room‐temperature stability threshold for t‐ZrO_2_ (≈30 nm), the metastable t‐phase could not be retained during cooling, leading to a complete t→m phase transformation [29]. A detailed discussion on the metastability of t‐ZrO_2_, crystallite growth behavior, and the associated phase transformation is provided in Figure 2a and Note S1.

The 0.5ZL NFMs exhibited a pure tetragonal phase at 800°C (Figure 2e), with an average grain size of ∼15.7 nm. No La_2_O_3_ peaks were detected, and slight peak shifts toward lower angles suggested lattice expansion due to La^3+^ substitution. Upon calcination at 1000°C, the tetragonal structure remained well preserved, accompanied by enhanced crystallinity with an average grain size of ∼16.8 nm (Figure S7). The experimental results demonstrated that incorporating a controlled amount of La(NO_3_)_3_ stabilizer enabled La^3+^ ions to substitute Zr^4+^ ions within the ZrO_2_ lattice at elevated temperatures, forming a substitutional solid solution. This substitution induced lattice distortion and substantially reduced the driving force for the t→m phase transformation, thereby stabilizing the tetragonal phase at lower temperatures—even under ambient conditions. The details of crystal growth behavior and lattice parameters are shown in Figure 2b and Note S2.

The 1.5ZL NFMs exhibited a cubic ZrO_2_ (c‐ZrO_2_) phase upon calcination at 800°C (Figure 2e), suggesting effective stabilization of the high‐temperature metastable phase through enhanced lattice distortion and oxygen vacancy generation. However, when the calcination temperature was increased to 1000°C, the formation of a secondary pyrochlore La_2_Zr_2_O_7_ (p) phase was observed (Figure 2f). This indicated that as the temperature rose and grain growth occurred (with the average grain size increasing from 14.3 to 21 nm), the solid solubility limit of La^3+^ in the ZrO_2_ lattice decreased, leading to a partial reaction between La^3+^ and ZrO_2_ to form the thermodynamically more stable pyrochlore phase. Rietveld refinement results showed that, compared to the 0.5 g doped sample, the diffraction peaks of 1.5ZL NFMs shifted toward lower angles, with the lattice parameter increasing from *a* = 5.164 Å to *a* = 5.204 Å and the unit cell volume expanding from 137.7 to 140.9 Å^3^. Notably, no diffraction peaks corresponding to free La_2_O_3_ were detected throughout the process, further confirming that La^3+^ predominantly existed in either a solid solution or compound state, governing the structural evolution of the material.

The mechanical properties of various ZrO_2_ NFMs were investigated through tensile tests, and their tensile stress‐strain curves are shown in Figure 2g. The 0.5ZL NFMs calcined at 800°C exhibited the optimal mechanical performance, with a fracture stress of approximately 1389 kPa at a strain of 7.27% and a Young's modulus of about 19106 kPa. As the temperature increased, grain growth occurred, resulting in a minor decline in the mechanical characteristics of 0.5ZL NFMs. The fracture stress reached 1040 kPa at a strain of 5.32%. Overall, the mechanical performance of 0.5ZL NFMs surpassed that of 1.5ZL NFMs (800°C‐1.5ZL NFMs: *σ* = 724 kPa, *ε* = 3.54%, *E* = 20452 kPa; 1000°C‐1.5ZL NFMs: *σ* = 547 kPa, *ε* = 2.64%, *E* = 20720 kPa). In contrast, pure ZrO_2_ NFMs exhibited the lowest mechanical performance (*σ* = 19 kPa, *ε* = 2.23%, *E* = 852 kPa) (Figure S8). This phenomenon occurred because tensile stress was generated near the tips of microcracks in the 0.5ZL NFMs containing the tetragonal phase under external forces, which helped to relieve the compressive stress acting on the tetragonal zirconia. Consequently, a layer of the tetragonal phase on the microcrack surface underwent transformation into the monoclinic phase. This phase transition resulted in a volume expansion of 3%–5% accompanied by shear strain, collectively generating compressive stress. This compressive stress not only counteracted the tensile stress caused by the external force, but also inhibited further phase transformation. Only the application of greater external forces could drive the continued phase transformation, allowing cracks to propagate and create new free surfaces until fracture occurred, thereby enhancing the fracture toughness and strength of the material [30]. Therefore, it was concluded that the addition of La(NO_3_)_3_ induced a gradual change in the phase composition of the sintered body. With the decrease in the monoclinic phase and the increase in the tetragonal phase, the toughening effect due to the phase transformation was strengthened, leading to an improvement in both the strength and toughness of the material. When the sintered body was entirely composed of the tetragonal phase, the material achieved maximum fracture toughness. However, when a certain amount of cubic phase appeared in the material, the fracture toughness decreased sharply due to the lower strength and toughness of the cubic phase and the reduced availability of transformable phases [31]. Under external stress, the propagation of the main crack in the matrix was less constrained by phase transformation, leading to a significant decline in material strength, particularly in fracture toughness. The fatigue performance of 0.5ZL NFMs calcined at 800°C was evaluated using tensile cyclic tests (Figure 2h). When the strain was 2.75%, the stress decreased by only about 6.67% after 100 tensile stress‐strain cycles, indicating that 0.5ZL NFMs possessed excellent flexibility, in sharp contrast to pure ZrO_2_ NFMs (Figure S9). Based on these findings, the 0.5ZL NFMs calcined at 800°C were selected as the substrate for subsequent MOF loading and referred to as ZL NFMs in the following sections.

#### 2.1.1 Construction of Superhydrophobicity and Heterojunctions on Ceramic NFMs

The superhydrophobic Zr‐based MOF heterojunction photocatalysts were loaded with high stability via a three‐step solvothermal synthesis. First, a seed layer was formed through strong coordination bonding between Zr ions at the metal centers of ZrO_2_ fibers and 2‐aminoterephthalic acid (ATA). Next, a secondary solvothermal synthesis was carried out on the seed layer by introducing ZrCl_4_ and organic ligands. The seed layer guided the directional heteroepitaxial growth of Zr‐based MOFs, enhancing interfacial binding strength via coordination bonds. Finally, CdS was loaded onto the MOF, and the surface was modified to be superhydrophobic (Figure 3a). This approach achieved robust loading of photocatalysts on ZrO_2_ nanofiber membranes. The samples were characterized by SEM and transmission electron microscope (TEM), with detailed morphological analyses of both pristine and modified MOFs provided in Figure S10. After seed layer growth and secondary growth, UiO‐66‐NH_2_ particles adhered tightly to the surface of ZL fibers, confirming the successful growth of MOF particles on the ceramic fibers (Figures 3b and S11). To compare the effects of seeding on fiber morphology, ZL NFMs were directly immersed in a solution of ZrCl_4_ and ATA for in situ deposition. It was observed that UiO‐66‐NH_2_ crystals did not completely and densely cover the surface of ZL fibers (Figure S12), indicating that ATA can directly react with Zr^4+^ in ZrO_2_. This reaction formed a seed layer that uniformly covered the fiber surface, thereby promoting the nucleation and growth of the crystals. The TEM analysis in Figure 3c clearly demonstrated the presence of nanoparticles on UiO‐66‐NH_2_ fibers. The lattice spacing of 0.34 nm observed in the high‐resolution transmission electron microscopy (HRTEM) image (Figure 3d), along with the corresponding fast Fourier transform pattern, was in excellent agreement with the (111) crystal plane of CdS, confirming the successful attachment of CdS to the MOF framework [32]. Simultaneously, energy‐dispersive x‐ray spectroscopy (EDS) analysis (Figure S13) confirmed the presence of Cd and S elements on the fiber surfaces. Furthermore, the chemical structure of the samples was analyzed using FTIR and XRD (Figure 3e, f), with the infrared and XRD spectra of pristine and modified MOFs materials provided in Figures S14 and S15. In both the FTIR and XRD spectra, all characteristic peaks of UC were observed in the ZLUC NFMs, confirming that UC had successfully grown on the surface of zirconia fibers.

**FIGURE 3:**
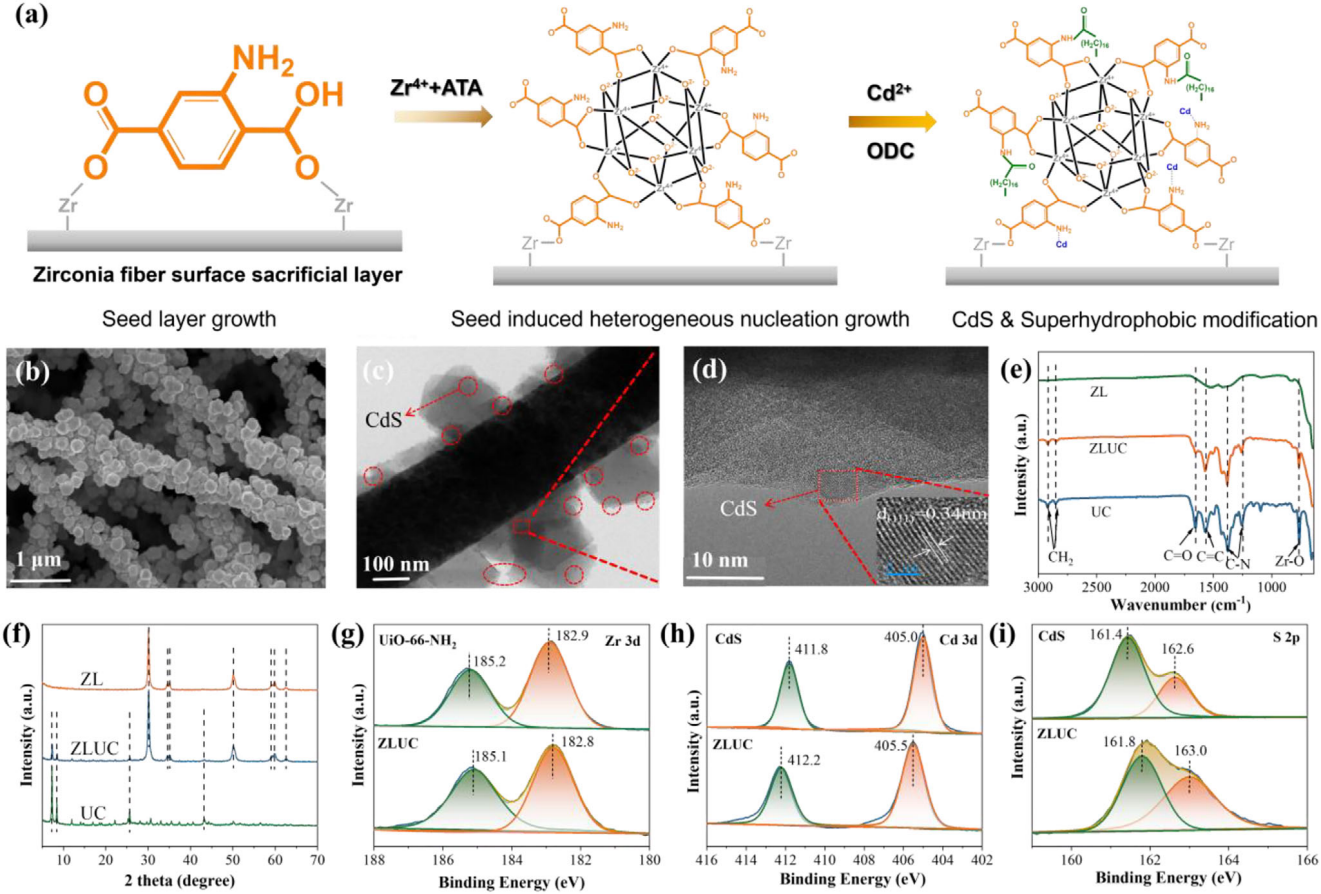
Fabrication and structural characterization of ZLUC NFMs. (a) Schematic illustration of the three‐step solvothermal construction of a superhydrophobic Zr‐MOFs/CdS heterojunction photocatalyst with high stability on ZrO_2_ ceramic nanofiber membranes via seed‐layer formation, oriented epitaxial growth, CdS loading, and superhydrophobic modification. (b) SEM images and (c) TEM images of ZLUC NFMs. The dark particles highlighted by red circles in the TEM image correspond to CdS. (d) HRTEM image of CdS on ZLUC NFMs. (e) FTIR patterns and (f) XRD patterns of multiple samples. The high‐resolution XPS spectra of (g) Zr 3d, (h) Cd, and (i) S.

X‐ray photoelectron spectra (XPS) analysis revealed the chemical states of elements in ZLUC NFMs. Compared with pristine UiO‐66‐NH_2_ and CdS, the Zr 3d peaks in the composite slightly shift toward lower binding energies, while the Cd 3d and S 2p peaks shift toward higher binding energies, suggesting possible interfacial electron transfer from CdS to UiO‐66‐NH_2_ or interfacial hole transfer from UiO‐66‐NH_2_ to CdS (Figure 3g‐i). In other words, the electron cloud density increased on UiO‐66‐NH_2_ while it decreased on CdS, showing that UiO‐66‐NH_2_ and CdS had formed a type‐II heterojunction in the ZLUC NFMs. Under the influence of the internal electric field, electrons in the conduction band (CB) of CdS transferred rapidly to the CB of UiO‐66‐NH_2_, while holes in the valence band (VB) of UiO‐66‐NH_2_ migrated in the opposite direction toward the VB of CdS. This charge transfer process is further supported by electrochemical studies, as will be shown in the following sections. Furthermore, because of the coordination relationship between Cd(II) and the amino groups [33], the intensity of the N 1s spectrum in UiO‐66‐NH_2_ (Figure S16) shifted toward the higher energy region after the heterojunction with CdS was formed.

### 2.2 Conversion to a Superhydrophobic State Exhibiting Excellent Water Repellency

Surface wettability significantly influences the performance of MD. Insufficient hydrophobicity (CA < 90°) can lead to membrane pore wetting and a decline in permeate flux. The ZL NFMs exhibited hydrophilicity (CA of 64°; Figure 4a), which was attributed to the abundant hydroxyl groups and oxygen vacancies on the ZrO_2_ surface that readily formed hydrogen bonds with water molecules. The CAs of the NFMs with MOF growth and superhydrophobic modification, but without CdS loading (ZLU), as well as ZLUC, increased significantly to 153° and 152°, respectively, confirming their superhydrophobicity. The improved hydrophobicity was attributed to the amidation reaction between the amino groups of UiO‐66‐NH_2_ and stearoyl chloride, which grafted hydrophobic stearoyl chains (C_17_H_35_‐) onto the surface. Additionally, the loading of CdS did not affect the superhydrophobicity of the membranes. The surface roughness of electrospun fiber membranes played a critical role in determining their wettability. Confocal laser scanning microscopy (CLSM) revealed that the ZLUC NFMs exhibited a significantly higher surface roughness (3.4 µm) compared to the pristine zirconia‐based ZL NFMs (1.5 µm) (Figure 4b, c). This pronounced increase in roughness was attributed to the hierarchical micro/nanostructure formed by the in situ growth of UiO‐66‐NH_2_ on ZL fibers. Such dual‐scale roughness‐microscale fiber entanglement and nanoscale MOF‐synergistically contributed to the superhydrophobicity (CA >150°) by trapping air pockets and reducing solid‐liquid contact area, consistent with the Cassie‐Baxter model (Figure 4d).

**FIGURE 4:**
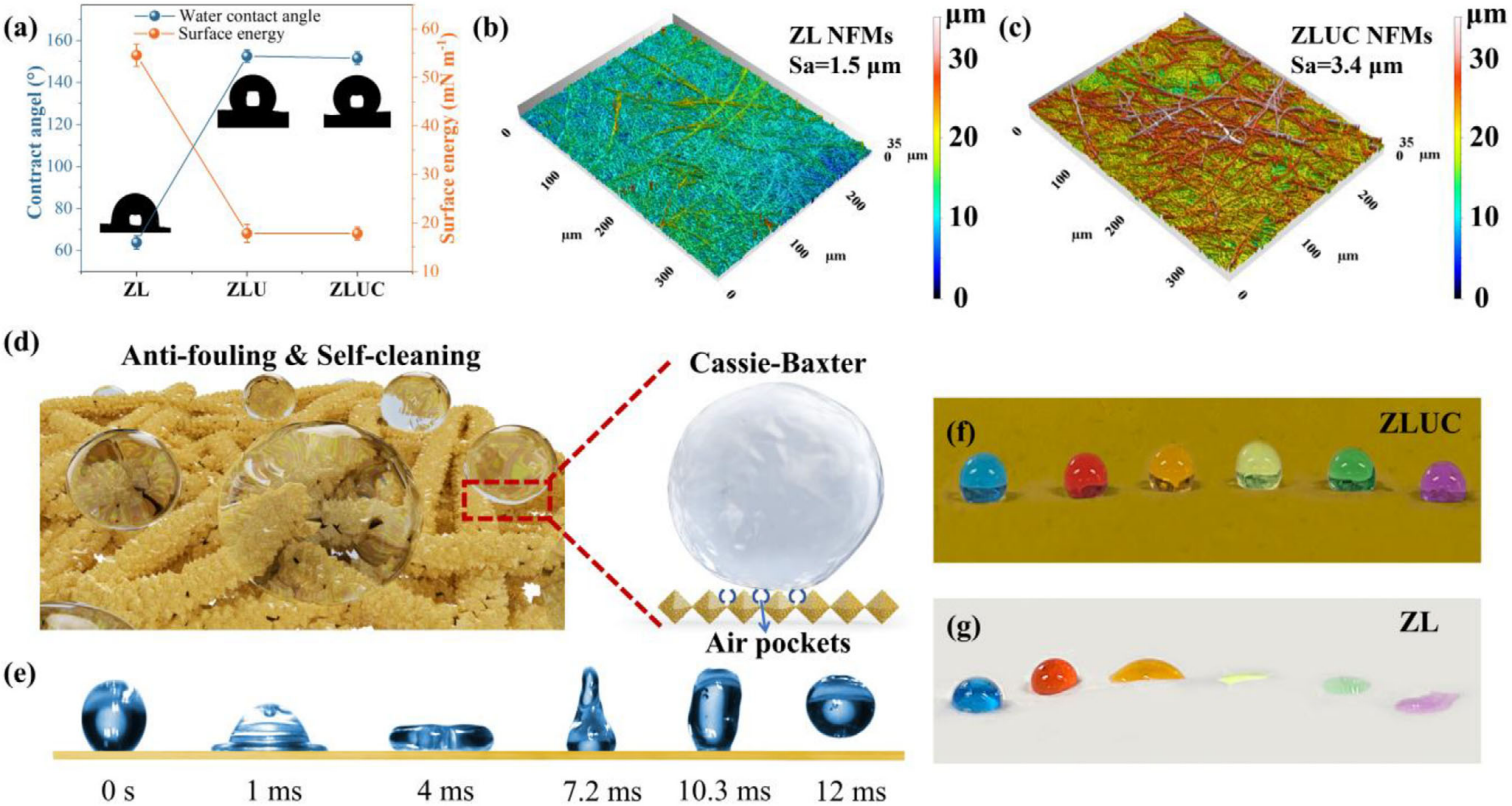
The hydrophobicity and stability of the membrane. (a) The water contact angle (CA) measurements of the NFMs, accompanied by their surface energy characteristics. Three‐dimensional surface morphology of (b) ZL and (c) ZLUC NFMs revealed by CLSM, with chromatic scale representing elevation differences. (d) Schematic illustration of the anti‐wetting performance of nano‐concave‐convex structured membrane surfaces. (e) Water droplet (0.5 mL) bouncing behavior on ZLUC NFMs, captured by a high‐speed camera. Optical images of various solvent droplets on (f) ZLUC and (g) ZL NFMs. From left to right: deionized water, 10% and 20% ethanol aqueous solutions, and 0.1, 0.3, and 0.5 mM sodium dodecyl sulfate (SDS) aqueous solutions.

The water rebound phenomenon is another hallmark of strong hydrophobicity. The impacting droplet did not penetrate the superhydrophobic surface of the ZLUC NFMs; instead, it rebounded into the air within 12 ms and underwent multiple bounces before finally settling (Figure 4e). This exceptional hydrophobic behavior originated from the low surface energy and hierarchical roughness of the membrane's topological structure. Furthermore, ZLUC NFMs exhibited a water droplet rolling angle of 4° (Figure S17), enabling droplets to roll off easily, which demonstrated their remarkable low‐adhesion superhydrophobic properties. Combining an ultra‐high CA of 152°, a low rolling angle of 4°, and the nanoscale rough morphology revealed by SEM images, the surface wetting behavior conformed to the Cassie‐Baxter model, indicating the presence of an air cushion effect between the droplet and the fibers. This characteristic not only ensures the material's anti‐wetting performance in MD, but also provides an efficient gas‐liquid‐solid triphase contact interface for photocatalytic reactions. Moreover, ZLUC NFMs also demonstrated excellent repellency toward low surface tension substances (Figure 4f), maintaining spherical droplets when ethanol and SDS solutions were deposited. In contrast, the pristine ZL NFMs (CA = 64°) exhibited hydrophilic properties (Figure 4g).

Abrasion tests were conducted to assess the mechanical durability of the ZLUC NFMs. The ZLUC coating maintained good super hydrophobicity within the initial 400 cm of wear, followed by a slight decline (Figure S18). Even after 1000 cm of abrasion, the coating retained notable hydrophobicity, with a CA of 150.2°. To verify the reinforcing effect of the seed layer on the coating's wear resistance, comparative tests were conducted on a ZLUC hydrophobic coating fabricated without the seed layer. As shown in the inset of Figure S18, its CA dropped below 114.1° under the same conditions. The poor durability of the seed‐free coating originated from the weak interfacial adhesion of MOFs, which were merely physically deposited on the ceramic fibers and thus were prone to detachment during abrasion. These results confirmed the critical role of the seed layer in constructing mechanically robust superhydrophobic surfaces.

### 2.3 Research on H_2_ Production and Desalination Performance

Designing an integrated membrane capable of simultaneous freshwater purification and hydrogen evolution is pivotal to advancing coupled photocatalytic and thermally‐assisted membrane distillation (PTMD) systems [34]. To fulfill the dual‐functional requirements, an ideal PTMD membrane must possess: (1) High porosity and excellent interfacial anti‐wetting ability to ensure sustained water vapor transport; (2) efficient broadband solar absorption and charge separation capability to support photocatalytic hydrogen evolution; and (3) robust chemical stability to withstand harsh operational conditions. Accordingly, ZLUC NFMs were designed as flexible ZrO_2_ ceramic nanofiber membranes integrated with superhydrophobic UiO‐66‐NH_2_/CdS heterojunctions. The ZrO_2_ nanofiber matrix provides mechanical robustness and irradiation resistance, while the porous architecture facilitates efficient vapor transport. The superhydrophobic modification, together with the construction of a UiO‐66‐NH_2_/CdS heterostructure, endows the membrane with superior charge separation efficiency and interfacial anti‐wetting capability. The MD‐relevant properties of ZLUC NFMs were quantified and summarized in Table S1. These features provide interconnected vapor pathways, sufficient resistance to liquid intrusion, and low conductive heat loss during VMD. At the microscopic level, the coupled PTMD/H_2_ evolution process proceeds through a dynamic gas–liquid–solid interface rather than a fully wetted catalyst surface or a dry vapor‐phase reaction. The superhydrophobic ZLUC surface maintains a Cassie–Baxter state that prevents bulk saline water from flooding the membrane pores and deeply penetrating the CdS‐containing catalytic layer, thereby preserving VMD selectivity and mitigating aqueous corrosion. However, this hydrophobic layer is not a dense encapsulation barrier: The UiO‐66‐NH_2_/CdS heterojunction remains exposed on the porous nanofiber surface, where local liquid menisci and adsorbed/condensed interfacial water can provide water/proton equivalents to the active sites. Under VMD operation, continuous water‐vapor transport replenishes this hydrated interfacial region, while dissolved Na_2_S/Na_2_SO_3_ sacrificial agents are supplied through liquid‐contacting meniscus regions. The generated H_2_ then desorbs into adjacent gas pockets and is removed through the vacuum‐assisted vapor/gas pathway, reducing bubble shielding and mass‐transfer resistance. Thus, the superhydrophobic interface balances catalyst protection, reactant accessibility, vapor transport, and gas‐product release within one membrane architecture [35, 36].

The membrane performance was evaluated using a customized VMD system specifically adapted for PTMD applications. Unlike direct contact MD, VMD generates a distinctive negative pressure that draws produced gases from the feed side to the permeate side, making it particularly suitable for cogeneration systems involving both gaseous and liquid products [37]. Figure 5a illustrates the PTMD setup designed for simultaneous freshwater and photocatalytic H_2_ evolution. ZLUC NFMs were mounted on a porous support featuring a transparent window at the top. A xenon lamp provided solar irradiation at a fixed distance with an intensity of one sun (1000 W m^−2^). To minimize light attenuation caused by water scattering, the thickness of the feed solution above the membrane surface was maintained at 2 mm. The feed solution was continuously circulated over the membrane at 10 mL min^−1^ using a peristaltic pump, with additional external heating supplied. The permeate chamber was evacuated by a vacuum pump, where the vapor condensed. The generated hydrogen was then transferred to a gas chromatography (GC) system for yield measurement.

**FIGURE 5:**
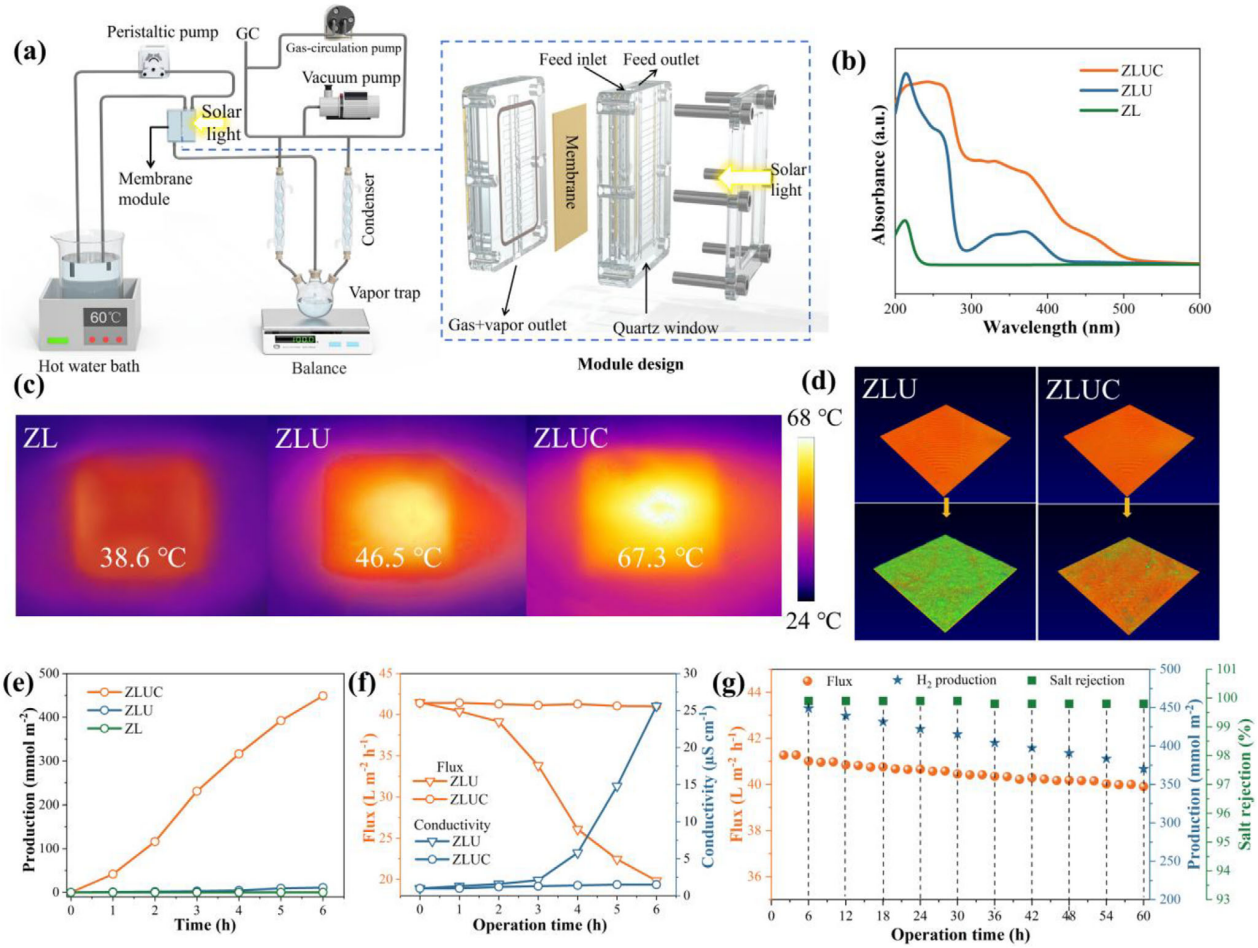
Membrane distillation coupled with photocatalytic hydrogen evolution: Performance evaluation and antifouling mechanism analysis. (a) Schematic illustration of PTMD water‐hydrogen cogeneration system, with the customized membrane chamber shown in the box on the right. Characterization of NFMs: (b) UV‐visible absorption spectra. (c) Infrared thermal image of the surface temperature of NFMs after being exposed to 1‐sun illumination for 15 min. (d) The OCT images of the employed NFMs surface after PTMD testing. The greater the green intensity, the more surface contaminant adhesion is indicated. (e) Hydrogen evolution performance of NFMs (0.2 M Na_2_S/Na_2_SO_3_ as sacrificial agent). (f) PTMD performance of ZLU and ZLUC NFMs, where ZLU refers to the superhydrophobic MOF membrane without CdS growth. (g) The long‐term stability of ZLUC NFMs (one sun illumination, with cycles repeating every 6 h for a total of 10 cycles).

As the PTMD process involves simultaneous solar‐driven water splitting for hydrogen evolution, it is essential to implement effective spectral management to maximize solar utilization efficiency, thereby enhancing the overall performance of the system. The optical properties of the prepared NFMs were evaluated by UV–vis spectroscopy. As shown in Figure 5b, the ZLUC NFMs exhibited enhanced absorption capabilities, particularly in the visible light region, due to the incorporation of CdS, which facilitated light absorption and the generation of photo‐excited electron‐hole pairs. This improvement enabled the ZLUC fibers to utilize a broader spectrum of solar energy compared to the ZLU NFMs. Additionally, the UV–vis reflectance spectra (Figure S19) revealed that the ZLUC fibers maintained low reflectance across the measured spectrum, which was attributed to their porous structure and the uniform distribution of photoactive materials. These properties collectively contributed to the efficient solar energy conversion required for both MD and photocatalytic hydrogen evolution.

The light‐induced thermal response of NFMs was characterized using an infrared camera. As shown in Figure 5c, under irradiation, ZLUC reached an equilibrium temperature of 67.3°C—significantly higher than pristine ZL (38.6°C) and ZLU NFMs (46.5°C), and markedly exceeding non‐irradiated controls (Figure S20). This temperature rise was attributed to the heterojunction's partial conversion of absorbed light energy into heat. The localized heating may provide two auxiliary benefits: (1) A marginal increase in the transmembrane temperature gradient, potentially enhancing vapor flux, and (2) thermal activation of CdS photocatalytic activity, which could accelerate charge carrier separation and hydrogen evolution kinetics [38, 39]. Additionally, the low thermal conductivity of ZrO_2_ fibers (0.5–1.5 W m^−1^ K^−1^) effectively minimized heat loss, enabling the system to maintain long‐term stability while achieving high solar energy utilization efficiency [40].

Under simulated conditions, the anti‐fouling performance of ZLU and ZLUC NFMs was evaluated using model pollutants including methyl orange and NaCl. Optical coherence tomography (OCT) imaging in Figure 5d reveals superior anti‐fouling capability of ZLUC over ZLU, with higher green intensity indicating more severe contaminant adhesion. ZLU effectively repels the physical penetration of salts and organic foulants owing to its unique concave–convex surface topography. In contrast, ZLUC further minimizes organic contaminant attachment through photocatalysis driven by the UiO‐66‐NH_2_/CdS heterojunction under light irradiation. This process promotes charge separation and induces reductive cleavage of methyl orange adsorbed on the membrane surface. Such a dual‐mechanism strategy—combining physical shielding (stable Cassie–Baxter state) with chemical decomposition (heterojunction‐driven photocatalysis)—effectively prevents contaminant accumulation and wetting at membrane pores, positioning ZLUC NFMs as a promising candidate for treating high‐salinity organic wastewater with simultaneous hydrogen production. To further evaluate short‐term biofouling resistance under illuminated operation, algal‐fouling tests were conducted using an algae‐containing NaCl feed solution with an initial algal concentration of OD_680_ = 0.30. ZLU and ZLUC NFMs were tested under identical PTMD conditions. In the algae‐containing feed, ZLU showed a rapid flux decline from 40.75 to 13.400 L m^−2^ h^−1^ within 6 h, indicating severe algal‐deposition‐induced vapor‐transport blockage and wetting. In contrast, ZLUC maintained a stable average flux of 39.2 L m^−2^ h^−1^, corresponding to 95.3% flux retention, together with a salt rejection of 99.93% and low permeate conductivity of 6.8 µS cm^−1^ (Figure S21). Post‐operation 2D‐OCT imaging revealed only a thin and discontinuous algal deposition layer on the ZLUC surface, without obvious dense biofilm formation or deep foulant intrusion (Figure S22). These results suggest that the UiO‐66‐NH_2_/CdS‐containing ZLUC membrane can mitigate short‐term algal‐cell deposition under illuminated PTMD operation while preserving stable vapor transport.

The PTMD performance of NFMs was evaluated. As shown in Figure 5e, ZLUC delivered the highest hydrogen evolution of 448.9 mmol m^−2^ (74.8 mmol m^−2^ h^−1^), far exceeding that of ZLU (11.0 mmol m^−2^, 1.8 mmol m^−2^ h^−1^), whereas the pristine ZL NFMs exhibited no detectable hydrogen evolution activity. The dry gas collected downstream of the condenser showed an H_2_ purity of 98.6 vol%, confirming that H_2_ was the dominant gaseous product under the tested PTMD conditions. The apparent quantum yield and solar‐to‐hydrogen efficiency of ZLUC NFMs were calculated to be ∼0.26% and 0.49%, respectively, demonstrating efficient solar energy conversion for photocatalytic hydrogen evolution under monochromatic light (see Supporting Information for calculation details). In the MD test (Figure 5f), ZLUC demonstrated stable and high water flux (consistently maintained at ∼41.0 L m^−2^ h^−1^) with extremely low conductivity of the produced water (< 5 µS cm^−1^) during 6 h continuous operation, indicating its excellent anti‐wetting capability and desalination performance. In contrast, ZLU exhibited gradually declining water flux and significantly increasing conductivity (eventually exceeding 25 µS cm^−1^) over time, reflecting substantial membrane wetting and pollutant penetration. Toward a complete description of the thermodynamic driving force and solute selectivity in the VMD process, water‐vapor fugacity and ion‐specific rejection were analyzed (Table S2). The transmembrane driving force was expressed as Δ*f_w_*  =  *f*_*w*,*f*_ ‐ *f*_*w*,*p*_, where *f_w,f_* and *f_w,p_* are the feed‐side and permeate‐side water‐vapor fugacities, respectively. Under the present low‐pressure VMD conditions, the fugacity coefficient of water vapor is close to unity; therefore, fugacity was approximated by the activity‐corrected vapor pressure. In addition, the concentrations of Na^+^, Cl^−^, and sulfur‐containing species in the permeate were quantified by ion chromatography/inductively coupled plasma, and their individual rejection values were all above 99.99%. These results confirm that ZLUC NFMs operate through vapor‐selective transport rather than ion‐specific sieving, effectively preventing nonvolatile ion crossover during VMD.

To further distinguish illumination‐induced effects from the baseline membrane contribution, dark VMD control experiments were conducted for ZLU and ZLUC NFMs under thermally matched conditions (Figure S23). The tests were performed without solar illumination while maintaining the same feed composition, feed temperature of 60°C, feed flow rate, permeate‐side cooling condition, vacuum condition, and effective membrane area as those used in the illuminated PTMD experiments. ZLU and ZLUC exhibited comparable initial fluxes of 40.90 and 40.45 L m^−2^ h^−1^, respectively, with the same initial permeate conductivity of 1.0 µS cm^−1^, indicating similar baseline vapor‐transport behavior under the same thermal driving force. However, both membranes suffered from severe performance deterioration in the absence of illumination. After 6 h of dark VMD operation, the fluxes of ZLU and ZLUC decreased to 19.80 and 20.30 L m^−2^ h^−1^, respectively, while their permeate conductivities increased to 24.2 and 23.4 µS cm^−1^. The simultaneous flux decay and conductivity increase suggest that, without photocatalytic antifouling, organic contaminant accumulation and partial wetting gradually dominate the membrane performance. These dark‐control results indicate that the stable PTMD performance of ZLUC under illumination originates from the combined effect of photothermal enhancement of the vapor‐pressure driving force and illumination‐enabled photocatalytic antifouling, rather than from a stronger baseline superhydrophobic architecture.

The operational durability of ZLUC membranes was systematically evaluated in a PTMD system over 60 h of cyclic operation (10 cycles). As illustrated in Figure 5g, the membranes maintained a stable water flux of 39.9 L m^−2^ h^−1^ and salt rejection of 99.8% throughout testing, while hydrogen evolution exhibited a gradual decline from 448.9 mmol m^−2^ (initial cycle) to 370.2 mmol m^−2^ (final cycle), representing a 17.5% reduction in photocatalytic activity. This activity decline should be recognized as a limitation of the CdS‐containing photocatalytic layer under saline operation and is likely associated with chloride‐induced surface passivation/complexation of CdS active sites and possible partial CdS photocorrosion, which reduce accessible catalytic sites and hinder interfacial charge transfer during prolonged irradiation [41, 42, 43]. To further evaluate the operational and environmental stability of the CdS‐containing membrane, post‐operation structural and leaching analyses were performed after the 60 h cyclic PTMD test. The XRD pattern of the used ZLUC NFMs retained the characteristic diffraction features of the pristine membrane without obvious newly formed crystalline impurity phases, suggesting that the crystalline structure of the heterojunction was largely preserved after operation. SEM/TEM observations further confirmed that the hierarchical fibrous morphology and the anchored UiO‐66‐NH_2_/CdS coating remained intact, with no apparent coating delamination or structural collapse. In addition, the water contact angle changed only slightly from the initial 152° to post‐reaction 151.1°, indicating that the membrane maintained its superhydrophobic anti‐wetting surface after long‐term operation. Inductively coupled plasma (ICP) analysis was further conducted to quantify Cd release in both the feed and permeate streams. After 60 h operation, the Cd concentration in the final feed solution was 1.8 µg L^−1^, corresponding to 0.06% of the total Cd loading on the membrane, while the Cd concentration in the collected permeate was below the detection limit (0.05 µg L^−1^). These results demonstrate limited Cd leaching from the photocatalytic layer and no detectable Cd crossover into the distillate under the present laboratory PTMD conditions (Figures S24‐S27).

Finally, the salinity‐dependent PTMD performance of ZLUC NFMs was evaluated using NaCl solutions of 5800, 10000, 20000, and 35000 ppm. With increasing salinity, the water flux decreased from 40.8 to 32.6 L m^−2^ h^−1^, while the membrane maintained low permeate conductivity of 4.8–9.1 µS cm^−1^ and high salt rejection of 99.95%–99.98%. The H_2_ evolution rate decreased from 73.2 to 65.2 mmol m^−2^ h^−1^, indicating that ZLUC NFMs retain stable desalination and photocatalytic H_2_ evolution under seawater‐level NaCl conditions (Table S3). Compared with previously reported electrospun NFMs, the ZLUC NFMs with a special fiber surface structure show significant MD advantages (Table S4), while additionally enabling photocatalytic H_2_ evolution within the same membrane platform.

To better elucidate the charge‐transfer and H* adsorption behavior of the UiO‐66‐NH_2_/CdS heterojunction during photocatalytic H_2_ evolution, a molecular model of the UC composite was constructed (Figure S28), and DFT calculations were performed to simulate interfacial charge transfer and hydrogen adsorption processes. Figure 6a illustrates the charge density distribution, where yellow and cyan bubble regions represent electron accumulation and depletion, respectively, highlighting electron transfer from CdS to MOF. In the hydrogen evolution reaction, the hydrogen adsorption Gibbs free energy (Δ*G*_H*_) of an ideal catalyst should approach zero regardless of whether Δ*G*_H*_ is positive or negative. The high Δ*G*_H*_ value of CdS indicates its inferior catalytic performance for the hydrogen evolution reaction. Figure 6b displays the Δ*G*_H*_ values and their corresponding adsorption geometries. In ZLUC NFMs, H‐MOF‐CdS denotes hydrogen atoms adsorbed on the UiO‐66‐NH_2_ surface, while MOF‐CdS‐H represents those on the CdS surface. After coupling CdS with MOF, the Δ*G*_H*_ of the H‐MOF‐CdS composite (−0.584 eV) moves closer to zero compared to MOF‐CdS‐H (−0.717 eV) and pristine CdS (0.88 eV), suggesting that hydrogen evolution reaction preferentially occurs on the MOF surface of the composite. These calculations demonstrate that in ZLUC NFMs, electrons are initially photoexcited in CdS and subsequently migrate to the MOF side, where the MOF exhibits higher intrinsic catalytic activity, thereby enhancing hydrogen generation.

**FIGURE 6:**
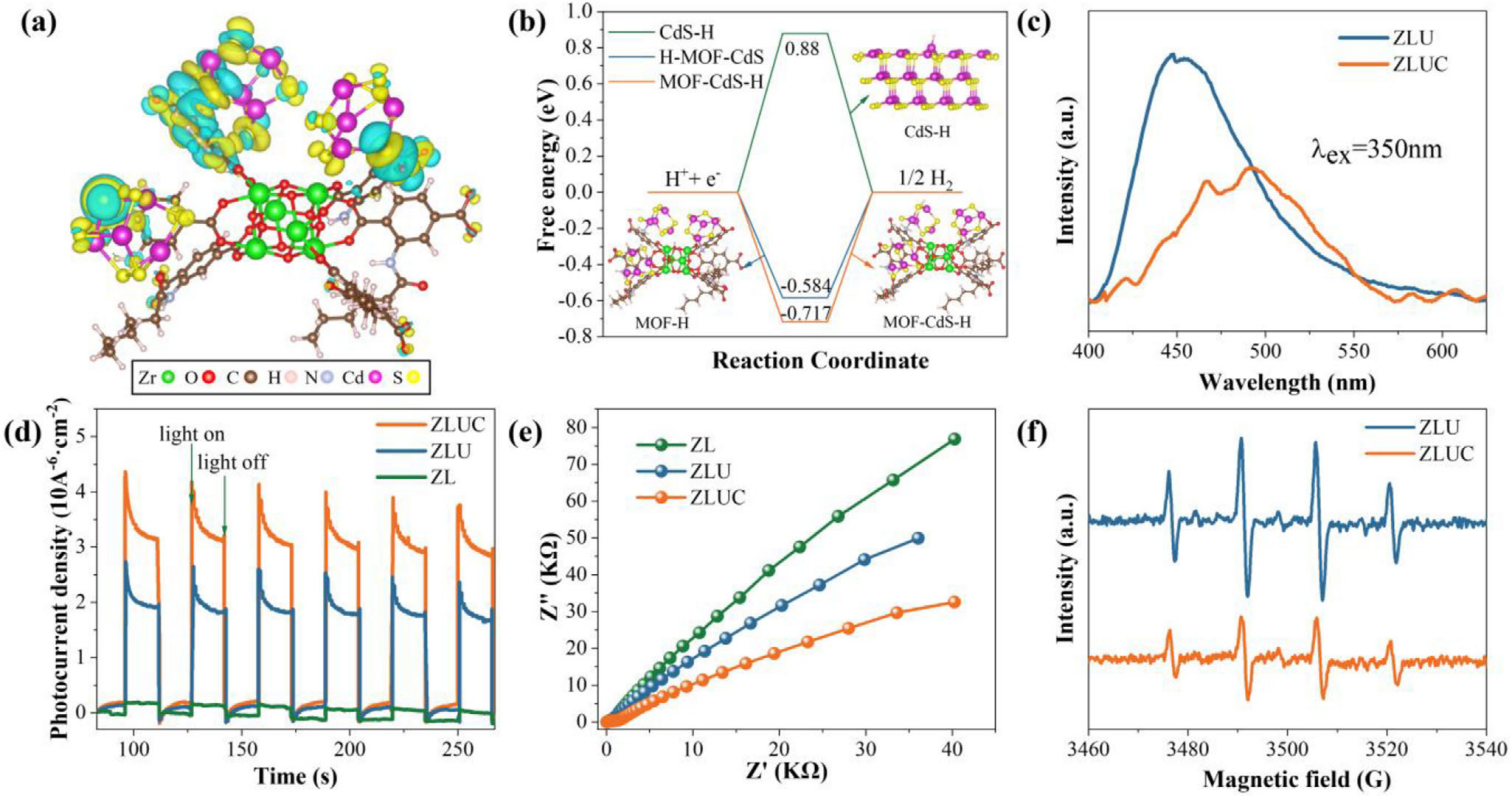
Density functional theory (DFT) computations and electrochemical performance measurements. (a) The charge density distribution across the UC heterojunction obtained from DFT computations. (b) The adsorption free energy (Δ*G*_H*_) of hydrogen atoms and their associated structural configurations calculated using DFT. (c) PL spectra, (d) TP, and (e) EIS of the NFMs. (f) EPR spectra of DMPO‐·OH for ZLU and ZLUC NFMs under visible light illumination.

The charge transfer pathway was elucidated through Mott‐Schottky plots (Figure S29a, c). Both types of semiconductors exhibited characteristics of typical n‐type semiconductors, as evidenced by their positive slopes. The flat‐band potentials of UiO‐66‐NH_2_ and CdS at various frequencies (500, 1000, and 1500 Hz) were determined from the horizontal intercepts, yielding values of −0.60  and −0.72 V versus the normalized hydrogen electrode (NHE), respectively. Since the flat‐band potentials approximately correspond to the lowest unoccupied molecular orbital (LUMO) level of UiO‐66‐NH_2_ and the CB level of CdS, they can be utilized to estimate the LUMO level of UiO‐66‐NH_2_ and the CB energy of CdS [44]. Considering that the LUMO potential of UiO‐66‐NH_2_ and the CB of CdS are more negative than the reduction potential of H^+^/H_2_ (2H^+^+2e^−^→H_2_, −0.41 V vs NHE), the reduction of H^+^ can theoretically be achieved [45, 46]. Additionally, the direct band gaps of UiO‐66‐NH_2_ and CdS were determined to be 3.00 eV and 2.51 eV, respectively, based on Tauc plot analysis (Figure S29b, d). Accordingly, calculations indicated that the highest occupied molecular orbital (HOMO) level of UiO‐66‐NH_2_ and the VB level of CdS relative to NHE are 2.40 and 1.79 V, respectively. Notably, the energy band alignment between CdS and UiO‐66‐NH_2_ is well‐matched, which supports the formation of a heterostructure and enhances the efficiency of charge transfer.

To assess charge transfer and separation efficiencies, photoluminescence (PL), transient photocurrent (TP), and electrochemical impedance spectroscopy (EIS) analyses were performed. The PL spectrum (Figure 6c) revealed suppressed fluorescence and a red‐shifted emission peak for ZLUC NFMs, indicating reduced carrier recombination and effective heterojunction formation upon CdS loading. Transient photocurrent responses (Figure 6d) showed that ZLUC exhibited the highest photocurrent among all samples, reflecting enhanced charge separation and prolonged carrier lifetime. EIS results (Figure 6e) further confirmed improved interfacial charge transfer, with ZLUC showing the smallest arc radius, consistent with the most efficient carrier separation enabled by the heterostructure.

The electron paramagnetic resonance (EPR) experiment further confirmed the charge transfer mechanism of ZLUC NFMs. The DMPO‐·OH EPR signal displayed four characteristic peaks with an intensity ratio of 1:2:2:1, arising from the oxidation of OH^−^ radicals into ·OH at an energy level of 1.99 eV [47]. Electrons flow from CdS to UiO‐66‐NH_2_ and holes migrate from UiO‐66‐NH_2_ to CdS if the UC heterojunction functions through a type‐II charge transfer mechanism. In this case, either no DMPO‐·OH signal or only a weak one would be detected. As shown in Figure 6f, compared with ZLU NFMs, the DMPO‐·OH peaks of ZLUC NFMs decreased, indicating that some holes on UiO‐66‐NH_2_ were transferred to the VB of CdS, which could not excite ·OH. The experimental results also confirmed the existence of a conventional type‐II heterojunction, consistent with the XPS analysis results mentioned earlier.

Based on XPS and EPR analyses, a photocatalytic hydrogen evolution mechanism is proposed for ZLUC NFMs. Under visible‐light irradiation, electrons and holes are generated in the LUMO/CB and HOMO/VB of UiO‐66‐NH_2_ and CdS deposited on ZL fibers, respectively. The more negative CB potential of CdS drives electron migration to the LUMO of UiO‐66‐NH_2_, achieving spatial charge separation that effectively inhibits charge recombination, prolongs carrier lifetime, and promotes proton‐coupled water reduction to H_2_. Meanwhile, the exceptional superhydrophobicity of ZLUC NFMs constructs a stable gas–liquid interface, repelling salt ions while enabling continuous water vapor transport. Thus, the heterojunction‐driven charge separation discussed here is coupled with VMD‐enabled vapor transport and H_2_ removal, enabling simultaneous desalination and photocatalytic H_2_ evolution within one membrane architecture.

### 2.4 Light‐Trapping and Bubble‐Release Mechanisms Enabled by the Three‐Dimensional Fibrous Architecture

To elucidate the structure‐optical‐activity relationship, planar control samples were prepared by coating the same loading of powder catalyst onto a PVDF substrate (PUC) of equal area and compared with the three‐dimensional fiber‐network‐supported sample (ZLUC). ZLUC exhibits a markedly higher H_2_ evolution yield (448.9 mmol m^−2^) than PUC (232.2 mmol m^−2^), underscoring the critical role of hierarchical architecture in solar energy utilization. To further understand the origin of this enhancement, finite‐difference time‐domain (FDTD) simulations were performed. Figure 7a, b illustrates the physical models used to calculate the optical absorption of the ZLUC and PUC structures, respectively. The FDTD results reveal that ZLUC generates highly non‐uniform and strongly enhanced localized electromagnetic fields throughout the fibrous network (Figure 7c). Within this architecture, incident photons undergo multiple scattering, coupling, and light‐trapping processes, which effectively prolong the optical path length and increase the probability of photon–active site interactions. In contrast, PUC shows surface‐confined electromagnetic fields with dominant specular reflection and negligible internal penetration, resulting in limited light‐harvesting efficiency (Figure 7d). A further comparison of the simulated absorption spectra (Figure 7e) reveals that ZLUC demonstrates higher absorption efficiency across a broader spectral range, particularly in the longer‐wavelength region. This can be primarily attributed to the multi‐level scattering effect induced by the three‐dimensional fibrous network structure, which significantly increases the effective optical path length of photons, thereby enhancing the probability of interaction between light and catalytically active sites. These results collectively demonstrate that the three‐dimensional, multi‐level scattering architecture of ZLUC enables efficient light trapping and photon utilization, providing a direct mechanistic basis for its superior photocatalytic performance.

**FIGURE 7:**
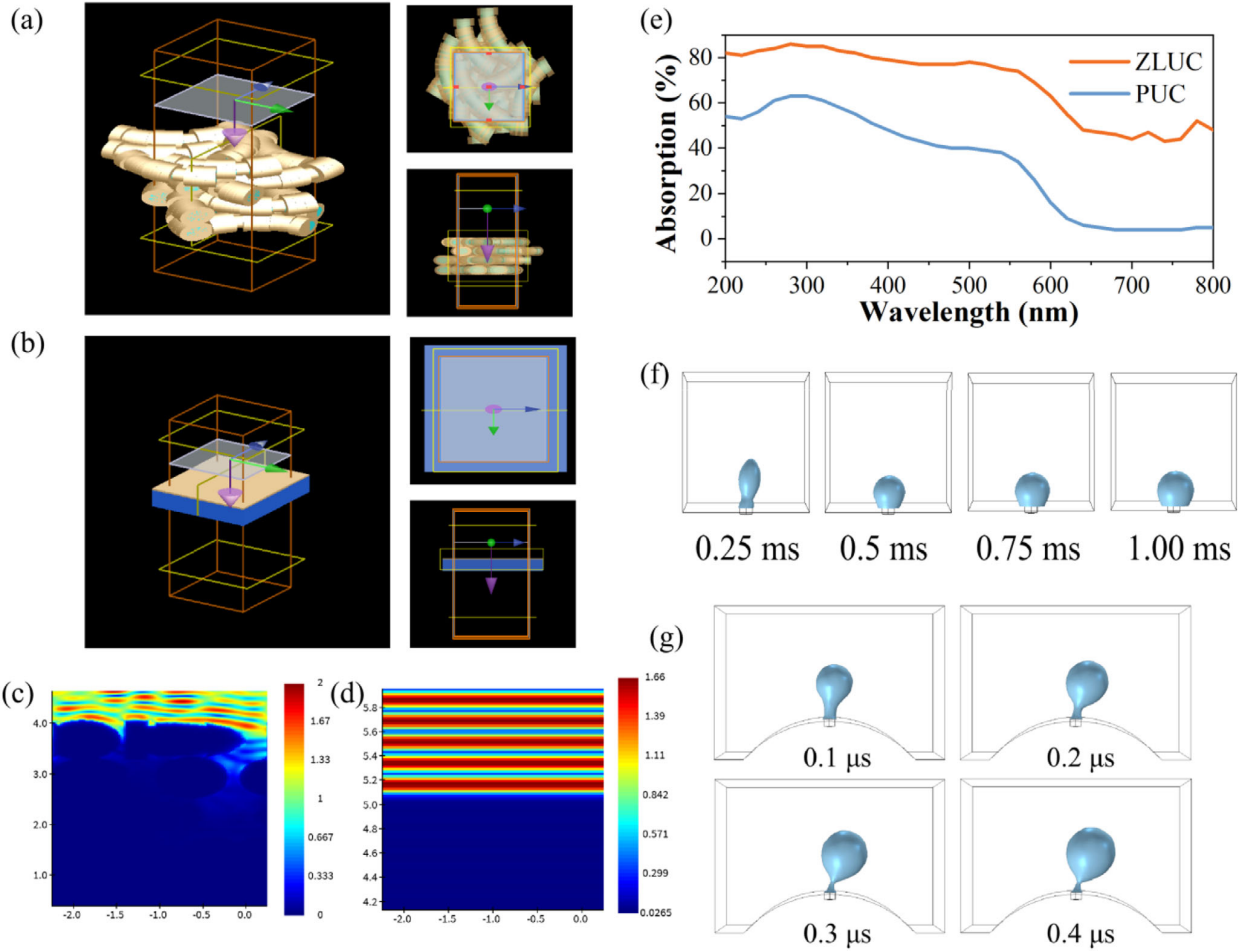
Light harvesting and bubble‐release behavior of ZLUC and PUC architectures. FDTD‐based physical models for calculating the optical absorption of (a) ZLUC and (b) PUC structures. Electric field distribution of the (c) ZLUC and d) PUC structures at 400 nm wavelength. (e) Comparison of absorption spectra between ZLUC and PUC. Transient CFD simulation of bubble nucleation, growth, and detachment on the (f) PUC and (g) ZLUC surface.

Beyond the light‐harvesting advantage revealed by FDTD simulations, gas–liquid mass transfer and product desorption are also crucial for determining photocatalytic performance. To elucidate the bubble evolution behavior on different structures, transient CFD simulations were conducted to visualize the nucleation, growth, and detachment of bubbles on the dense planar PUC and the fibrous ZLUC surfaces. As shown in Figure 7f and g, bubbles on PUC rapidly grow and remain attached to the dense surface, forming a shielding layer that hinders light penetration and reactant transport. In contrast, bubbles generated on the ZLUC fibers grow and detach rapidly, escaping through continuous gas pathways along the fibrous network. These results demonstrate that the three‐dimensional fibrous architecture promotes rapid bubble release and efficient gas transport, thereby enhancing gas–liquid mass transfer and suppressing bubble‐induced light shielding. Taken together, the DFT calculations, band‐structure analysis, and comprehensive photoelectrochemical characterizations confirm that the UiO‐66‐NH_2_/CdS heterojunction promotes spatial charge separation and interfacial electron transfer for H_2_ evolution, while the FDTD and CFD simulations further show that the three‐dimensional fibrous architecture enhances light harvesting and accelerates gas‐product removal.

## 3 Conclusion

In summary, this work presents a scalable and sustainable strategy for the integrated management of water and energy resources through a solar‐driven system. The ZLUC NFMs demonstrate outstanding performance for simultaneous saline‐water desalination and hydrogen evolution, achieving a hydrogen evolution rate of 74.8 mmol m^−2^ h^−1^ and a stable water flux of 41.0 L m^−2^ h^−1^, benefiting from the synergistic coupling of superhydrophobic membrane distillation and photocatalytic processes. Meanwhile, FDTD and CFD simulations reveal that the three‐dimensional fibrous architecture enhances light harvesting while facilitating rapid desorption and transport of gaseous products, thereby improving interfacial gas–liquid mass transfer during the reaction. The membrane's robust mechanical properties, a high salt rejection of 99.95%, and excellent antifouling capability combined with long‐term operational stability retaining over 80% of its initial photocatalytic activity after 60 h make it a promising candidate for practical applications in water treatment and renewable energy generation. It should be noted; however, that the present H_2_ evolution tests were conducted in a model saline feed containing Na_2_S/Na_2_SO_3_ as sacrificial hole scavengers. Therefore, the current system should be regarded as a proof‐of‐concept platform for integrated desalination and photocatalytic H_2_ evolution rather than a reagent‐free seawater‐to‐hydrogen process. Future work should focus on replacing sulfide/sulfite sacrificial agents with benign or waste‐derived electron donors, developing sacrificial‐agent‐free operation through efficient water oxidation, and further optimizing heterojunction architectures for broader solar‐spectrum utilization and long‐term environmental compatibility. Overall, this study provides a materials‐design framework for multifunctional membrane systems that integrate separation, catalysis, and interfacial transport for water–energy co‐generation.

## Author Contributions

**Pengfei Lin**: Methodology, investigation, and writing – original draft. **Xiaochen Lu**: Methodology. **Jiawei Sun**: Writing – review & editing. **Shuai Guo**: Writing – review & editing. **Xinping He**: Visualization. **Chunhai Yi**: Methodology and supervision. **Zhenyao Wang**: Writing – review & editing. **Jiaxin Guo**: Conceptualization, investigation, supervision, and writing – original draft. **Swee Ching Tan**: Methodology and writing – review & editing. **Alicia Kyoungjin An**: Conceptualization, visualization, supervision, and writing – original draft.

## Conflicts of Interest

The authors declare no conflicts of interest.

## Supporting information




**Supporting File**: adma74777‐sup‐0001‐SuppMat.docx.

## Data Availability

All data supporting the findings of this study are available in the article, the Supplementary Information, and the source data file are provided with this paper.
