# Supplementary material for: Hierarchical Flexible Ceramic Nanofiber Membrane for Simultaneous Membrane Distillation and Photocatalytic Hydrogen Evolution
Source: Adv Mater. 2026 Aug 24;38(59):e74777. doi: 10.1002/adma.74777 (PMC13689893; doi:10.1002/adma.74777)
Supplement: Supplementary file 1 [file ADMA-38-e74777-s001.docx]

**Supporting Information**

**Hierarchical Flexible Ceramic Nanofiber Membrane for Simultaneous Membrane Distillation and Photocatalytic Hydrogen Evolution**

Pengfei Lin^1, 2^, Xiaochen Lu^1^, Jiawei Sun^3^, Shuai Guo^4^, Xinping He^1^, Chunhai Yi^1^, Zhenyao Wang^5^, Jiaxin Guo^1,*^, Swee Ching Tan^4,*^ and Alicia Kyoungjin An^3,*^

^1^ State Key Laboratory of Fluorine & Nitrogen Chemicals, School of Chemical Engineering and Technology, Xi’an Jiaotong University, Xi’an 710049, China

^2^ Laoshan Laboratory, Qingdao 266237, China

^3^ Department of Chemical and Biological Engineering, The Hong Kong University of Science and Technology, Clear Water Bay, Kowloon, Hong Kong, 999077, China

^4^ Department of Materials Science and Engineering, National University of Singapore, 9 Engineering Drive 1, Singapore 117574, Singapore

^5^ Department of Environmental Science and Engineering, Macau University of Science and Technology, Macau SAR 999078, China

**^*^ Co-corresponding authors:** Jiaxin Guo (jxguo@xjtu.edu.cn), Swee Ching Tan (msetansc@nus.edu.sg) and Alicia Kyoungjin An (Alicia.kjan@ust.hk)

**Methods**

**Materials and chemicals**

All reagents were purchased commercially and used in their original form without further purification.

**Sample preparation**

**Synthesis of ZLP NFMs**

First, 9 mL of zirconium acetate (Zr(OAc)_4_) was used to separately dissolve 0.5 g and 1.5 g of lanthanum nitrate hexahydrate (La(NO_3_)_3_·6H_2_O), respectively. The electrospinning precursor was then prepared by adding 0.25 g of PVP to each aforementioned solution and vigorously stirring it for 6 hours at room temperature. The resulting samples were labeled as (X)ZLP, where X = 0.5 or 1.5, based on the weight of La(NO_3_)_3_·6H_2_O added to the composite. The precursor was subsequently loaded into the electrospinning apparatus and processed with a feed rate of 1 mL h^-1^. The distance between the needle tip and the collector was maintained at 15 cm while applying a high voltage of 25.0 kV. Aluminium foil served as the collecting substrate for the ZLP composite fibers. The resulting membrane was then dried at 60 °C overnight to obtain (X)ZLP NFMs. In addition, the one without La(NO_3_)_3_·6H_2_O was referred to as ZP NFMs.

**Preparation of (x)ZL NFMs**

The prepared (X)ZLP nanofiber membranes were placed in a muffle furnace. The heat treatment temperature was increased from room temperature to 800 °C or 1000 °C at a rate of 2 °C min^-1^, maintained at this temperature for 120 minutes, and then cooled to room temperature inside the furnace. Finally, the (X)ZL nanofiber membranes were obtained. In addition, ZP NFMs were referred to as ZrO_2_ NFMs after calcination.

**Preparation of seed layer NFMs**

The 0.5ZL NFMs were submerged in a synthesis solution composed of 1 part ATA, 10.5 parts acetic acid (HAc), and 474 parts N,N-dimethylformamide (DMF) by mass. To put it briefly, 50 mL of DMF was used to dissolve 0.1 g of ATA. Then, 1 mL of HAc and a specific amount of 0.5ZL NFMs were added, and the mixture was sonicated for 15 minutes. The samples were heated for 12 hours at 120 °C in an oven, rinsed with water to remove any surface contaminants, and then vacuum-dried for 12 hours at 60 °C.

**Preparation of pristine ZLU NFMs**

The seed-layer NFMs were immersed in a precursor solution with a mass composition of 1.8 ATA : 2.3 ZrCl_4_ : 31.5 HAc : 474 DMF for synthesis. Specifically, 0.18 g of ATA and 0.23 g of ZrCl_4_ were dissolved in 50 mL of DMF, followed by the addition of 3 mL of HAc and an appropriate amount of seed-layer NFMs. The mixture was ultrasonicated for 15 min. Subsequently, the samples were transferred to an oven and heated at 120 °C for 24 h. After reaction, the membranes were thoroughly rinsed with water to remove surface impurities and then vacuum-dried at 60 °C for 12 h to obtain the ZLU NFMs. The preparation procedure for ZLU NFMs without a seed layer was identical to that described above, except that the seed-layer NFMs were replaced with 0.5ZL NFMs.

**Preparation of pristine ZLUC NFMs**

First, 2 mg of Cd(CH_3_COO)₂·2H_2_O was dissolved in 10 mL of ethanol under magnetic stirring, followed by the addition of ZLU NFMs. The mixture was ultrasonicated for 30 min to enhance the adsorption of Cd^2+^ and then heated at 60 °C for 10 min. Subsequently, 10 mL of an ethanol–water mixed solution (7:3, v/v) containing 1 mg of thioacetamide (TAA) was slowly added dropwise. The reaction was maintained at 60 °C for 2 h to ensure a controlled nucleation process of CdS. Afterward, the membranes were thoroughly rinsed with ethanol (three times) and hexane (once) to remove unreacted precursors, followed by vacuum drying to obtain the pristine ZLUC NFMs. The ZLUC NFMs without a seed layer were prepared using the same procedure as described above, except that the ZLU NFMs were replaced with seed-layer-free ZLU NFMs.

**Superhydrophobic modification**

The as-prepared ZLU or ZLUC NFMs were first dispersed in 100 mL of DMF containing an appropriate amount of triethylamine and continuously stirred in an ice bath. A mixed solution of tetrahydrofuran and octadecanoyl chloride (ODC) at a volume ratio of 1:1 was then added dropwise to the above dispersion. The resulting suspension was stirred at 45 °C for 24 h to promote the surface acylation reaction. After naturally cooling to room temperature, the modified membranes were collected and alternately washed with DMF and methanol three times to remove unreacted species. Finally, the products were vacuum-dried to obtain the superhydrophobic ZLU or ZLUC NFMs.

**Preparation of** **UiO-66-NH_2_**

ZrCl_4_ (0.233 g) and ATA (0.181 g) were dissolved in 50 mL of DMF, followed by 3 minutes of ultrasonic treatment. HAc (3.6 mL) was then introduced, and the ultrasonic process was repeated for another 3 minutes. The resulting uniform solution was poured into a 100-mL Teflon-lined autoclave and heated at 120 °C for 24 hours in an oven. After cooling to room temperature, the MOFs were activated by washing the samples sequentially with DMF and ethanol, and subsequently drying them under vacuum conditions.

**Preparation of CdS**

10 mL of ethanol was used to dissolve 92 mg of Cd(CH_3_COO)_2_·2H_2_O. Following ten minutes of heating the suspension to 80 °C, 10 mL of an aqueous TAA solution (containing 26 mg) was progressively poured dropwise into the flask. The mixture was vigorously stirred and heated at 80 °C for 30 minutes, followed by static heating at 80 °C for an additional 30 minutes. The precipitate that resulted was filtered and repeatedly washed with ethanol and water. It was then vacuum-dried for 12 hours at 60 °C.

**Preparation of UiO-66-NH_2_/CdS (UC)**

Generally, 10 mL of ethanol was used to dissolve 2 mg of Cd(CH_3_COO)_2_·2H_2_O. The solution was then heated to 80 °C for 10 minutes after 40 mg of UiO-66-NH_2_ powder was added and sonicated for 30 minutes. Following the injection of 10 mL of an aqueous solution containing 1 mg of TAA, the mixture was stirred for 30 minutes at 80 °C and then left to stand for an additional 30 minutes at the same temperature. After filtering and repeatedly washing the precipitate with water and ethanol, it was vacuum-dried for 12 hours at 60 °C. Then, superhydrophobic modification was performed as follows: The product and triethylamine (1:1 molar ratio) were dissolved in 12 mL of DMF under ice-bath conditions. To this solution, an equal-volume mixture of tetrahydrofuran and octadecanoyl chloride was gradually added. The reaction system was maintained at 45 °C with continuous stirring for 24 hours. Following natural cooling to room temperature, the product was isolated through centrifugation and subsequently subjected to three washing cycles using DMF and methanol.

**Preparation of PUC**

The UC powder with the same loading amount as that used in ZLUC NFMs was dispersed in ethanol and ultrasonicated for 10 min to obtain a colloidal suspension. The suspension was then drop-cast onto a PVDF substrate with the same effective area and dried in a vacuum oven at 60 °C.

### Membrane characterizations

#### Liquid contact angle (CA) and surface energy

The CA of DI water on the employed membranes was measured using a CA measuring system (KRÜSS, Germany). The CA value took the average of three repeated measurements. To determine the surface energy, the CA of diiodomethane was evaluated for all membranes. The membranes’ surface energies were investigated by double sessile drop measurement coupled with KRÜSS software.

**Hydrodynamic characterization of surface wettability**
**Water droplet bouncing test:** 0.5 mL water droplets were dropped from a needle 20 mm above the membrane surface, and the bouncing phenomenon of the water droplets on the membranes was recorded using a high-speed camera (Photron, frame rate of 3600 fps). The contact time, number, and height of water bounces were measured as indicators of membrane hydrophobicity, which indirectly reveal the contact area between the droplets and the membrane surface.

**Water droplet rolling test:** The rolling behavior of water droplets was quantitatively evaluated by depositing 0.5 mL droplets onto the tilted membrane surface using a microsyringe. The rolling angle was determined as the minimum inclination angle at which droplets completely detached from the surface, measured via a high-precision goniometer (DataPhysics OCA50, accuracy ± 0.5°). For statistical significance, tests were repeated at five distinct locations on each sample.

**Optical coherence tomography (OCT) imaging**
OCT was used as a non-destructive imaging technique to visualize the hydrated fouling layer and surface contaminant distribution on the membranes after PTMD operation. OCT images were acquired using a spectral-domain OCT system (TEL321C1, Thorlabs, USA) equipped with ThorImage OCT software. This system allows both two-dimensional cross-sectional B-scan imaging and three-dimensional volumetric reconstruction.

Before OCT characterization, the membrane samples were carefully removed from the PTMD module and gently rinsed with the corresponding NaCl feed solution to remove loosely attached contaminants while preserving the hydrated fouling layer. The samples were then placed in a Petri dish and kept in a hydrated state during imaging. For 2D-OCT analysis, cross-sectional B-scan images were collected perpendicular to the membrane surface at randomly selected positions. For 3D-OCT analysis, a series of consecutive B-scans were acquired over the selected membrane area and reconstructed into a three-dimensional image using the built-in software. The OCT backscattering intensity was used to qualitatively evaluate foulant deposition on the membrane surface, where higher signal intensity indicates stronger surface contaminant accumulation.

**Membrane thickness, porosity, pore size distribution, LEP, and thermal conductivity**

The membrane thickness was measured using both cross-sectional SEM images and a digital micrometer (Mitutoyo, Japan) at ten randomly selected positions. The porosity of the membranes was determined by an ethanol uptake method. Briefly, the dry membrane was weighed as *m_d_*, immersed in ethanol to fully fill the pores, gently wiped to remove excess surface liquid, and then weighed as *m_w_*. The porosity was calculated according to:

$$\begin{aligned} \text{ε}\text{ }\text{= }\frac{\text{(}\text{m}_{\text{w}}\text{-}\text{m}_{\text{d}}\text{) / }\text{ρ}_{\text{ethanol}}}{\text{Aδ}}\text{ }\text{× 100\%}\# \end{aligned}$$

where *ρ*_ethanol_ is the density of ethanol, *A* is the effective membrane area, and *δ* is the membrane thickness. The pore size distribution and bubble-point pore size were measured using capillary flow porometry (CFP-1500-A, Porous Materials Inc., USA) with a fully wetting liquid. The liquid entry pressure (LEP) was measured using a custom-built dead-end pressure cell, in which the membrane was fixed between the feed and permeate chambers, and the feed side was filled with the model saline solution. The pressure was gradually increased until the first liquid droplet appeared on the permeate side. The corresponding pressure was recorded as the LEP. The effective thermal conductivity of the membrane was measured by the transient plane source (TPS) method using a thermal constants analyzer (TPS 2500 S, Hot Disk, Sweden) at room temperature.

**Characterization**

Thermogravimetric analysis (TGA) was performed using a Netzsch Thermoanalyzer STA 449 F5 apparatus at a heating rate of 20 °C min^−1^, spanning temperatures from 20 °C to 800 °C. X-ray diffraction (XRD) analysis was performed on a Shimadzu diffractometer equipped with Cu Kα radiation. At the same time, [Fourier transformation](https://www.sciencedirect.com/topics/chemistry/fourier-transform) infrared (FTIR) spectra were obtained using a Nicolet IS50 spectrometer. X-ray photoelectron spectra (XPS) measurements were conducted on a Thermo Scientific K-Alpha instrument, utilizing a monochromatic Al Kα source and a fixed analyzer pass energy of 50 eV. The morphology of catalysts and the produced membranes was examined using a scanning electron microscope (SEM, TESCAN MAIA3 LMH) equipped with an energy-dispersive X-ray spectroscopy (EDS) detector and a transmission electron microscope (TEM, Talos L120C G2). N_2_ adsorption-desorption isotherms were determined using a Beishide analyser. Confocal laser scanning microscopy (CLSM) was performed using an Olympus FV3000 microscope. The solution's metal content was quantified using inductively coupled plasma mass spectrometry (ICP-MS, Agilent 7900). UV–vis diffuse reflectance spectra were recorded using a PE Lambda 950 spectrophotometer equipped with a 150 mm integrating sphere. The photoluminescence (PL) spectra were obtained with an Edinburgh FLS9 spectrometer. Electron paramagnetic resonance (EPR) spectra were obtained utilising SPINSCAN X (Adani, Belarus) at ambient temperature.

**Performance evaluation**

PTMD water-hydrogen co-production was evaluated in a VMD setup. The membrane module was equipped with an optical quartz window at the top to allow illumination from a 300 W xenon lamp (PLS-SME400E H1, Beijing Perfectlight) fitted with an AM 1.5G filter. During PTMD testing, simulated solar irradiation was directed vertically onto the membrane at a fixed distance of 12 cm, with light intensity maintained at 1 kW m^-2^ via current adjustment. To minimize light scattering from the water layer, the gap between the membrane surface and the glass cover was precisely controlled at ~2 mm. The feed solution (0.1 M NaCl/Na_2_S/Na_2_SO_3_ and 10 mg L^-1^ methyl orange) was circulated through the module at a flow rate of  10 mL min^-1^ and maintained at 60 °C. Permeate-side cooling was achieved using cooling water (20 °C) flowing at 40 mL min^-1^, while desalinated water was collected for analysis. Hydrogen concentration was quantified at 60-min intervals using gas chromatography. The composition of the collected gas was analyzed after condensation of water vapor from the vacuum-side gas. Gas samples were collected using a gas-tight syringe and analyzed by gas chromatography equipped with a thermal conductivity detector using calibrated standard gases. The H_2_ purity was calculated on a dry-gas basis according to:

$$\text{H}_{\text{2}}\text{ }\text{purity }\text{=}\frac{\text{n}_{\text{H2}}}{\sum\text{n}_{\text{i}}}\text{ × 100\%}$$

where *n*_H2_is the molar amount of H_2_ and $\sum\text{n}_{\text{i}}$ is the total molar amount of detected dry gases.

Water flux (*J*, kg m^-2^·h^-1^) was calculated as:

$$\begin{aligned} \text{J}\text{ }\text{=}\text{ }\frac{\text{Δ}\text{m}}{\text{A}\text{·Δ}\text{t}}\# \end{aligned}$$

where ∆*m* (kg) is the weight of the collected water, *A* (m^2^) is the surface area of the membrane, and ∆*T* (h) is the operation time.

Salt rejection (*R* %) was calculated as follows:

$$\begin{aligned} \text{R}\text{ }\text{=}\text{ }\frac{\text{C}_{\text{f}}\text{ }\text{-}{\text{ }\text{C}}_{\text{p}}}{\text{C}_{\text{f}}}\text{ }\text{×}\text{ }\text{100\%}\#\text{ } \end{aligned}$$

where *C*_f_ and *C*_p_ (mS cm^-1^) represent the conductivity of the feed and permeate, respectively.

**Water-vapor fugacity and ion-specific rejection analysis**

The water-vapor fugacity difference across the membrane was calculated to describe the thermodynamic driving force for VMD. The feed-side water fugacity was estimated as:

$$\text{f}_{\text{w}\text{,}\text{f}}\text{ = }\text{a}_{\text{w}}\text{P}_{\text{w}}^{\text{0}}\text{(}\text{T}_{\text{f}\text{,}\text{m}}\text{)}$$

where $\text{a}_{\text{w}}$is the water activity of the feed solution and $\text{P}_{\text{w}}^{\text{0}}\text{(}\text{T}_{\text{f}\text{,}\text{m}}\text{)}$ is the saturated vapor pressure of pure water at the feed-side membrane temperature. The permeate-side water fugacity was calculated as:

$$\text{f}_{\text{w}\text{,}\text{p}}\text{=}\text{y}_{\text{w}\text{,}\text{p}}\text{P}_{\text{p}}$$

where $\text{y}_{\text{w}\text{,}\text{p}}$is the water vapor mole fraction on the permeate/vacuum side and $\text{P}_{\text{p}}$ is the permeate-side pressure. Under the low-pressure VMD conditions used in this work, the fugacity coefficient of water vapor was approximated as unity. Therefore, the transmembrane water-vapor driving force was expressed as:

$$\text{Δ}\text{f}_{\text{w}}\text{ }\text{= }\text{f}_{\text{w}\text{,}\text{f}}\text{ }\text{-}{\text{ }\text{f}}_{\text{w}\text{,}\text{p}}$$

The ion-specific rejection was calculated according to:

$$\text{R}_{\text{i}\text{ }}\text{= (1 - }\frac{\text{C}_{\text{i}\text{,}\text{p}}}{\text{C}_{\text{i}\text{,}\text{f}}}\text{) × 100\%}$$

where *C_i,f_* and *C_i,p_*are the concentrations of ion iii in the feed and permeate, respectively. Na⁺ was quantified by ICP-MS (Agilent 7900), while Cl⁻ and sulfur-containing anions were quantified by ion chromatography (Dionex ICS-6000).

**Algal-fouling test using algae-containing NaCl feed solution**
Chlorella vulgaris was used as a representative algal foulant. The algal suspension was added to the 0.1M NaCl solution, and the initial algal concentration was adjusted to an optical density of OD_680_ = 0.30, corresponding to a chlorophyll-a concentration of 120 μg L^-1^ and a cell density of approximately 1.0 × 10^6^ cells mL^-1^. Before the PTMD test, the feed solution was gently stirred to ensure homogeneous dispersion of algal cells.

The algal-fouling test was conducted in the PTMD system under the same operating conditions used for the desalination and photocatalytic H_2_ evolution tests, including the same effective membrane area, feed temperature, vacuum condition, flow rate, and simulated sunlight irradiation. The test was operated continuously for 6 h.

After the algal-fouling test, the membrane was removed from the module and gently rinsed with the corresponding NaCl feed solution to remove loosely attached algal cells. Two-dimensional optical coherence tomography (2D-OCT) was then employed as a non-destructive cross-sectional imaging technique to visualize the hydrated algal-fouling layer on the membrane surface.

The solar-to-vapor efficiency (*η*, %) was obtained using the following Equation:

$$\begin{aligned} \text{ƞ}\text{ }\text{=}\text{ }\frac{\text{J}\text{ }\text{× }\text{h}_{\text{LV}}}{\text{P}_{\text{light}}}\# \end{aligned}$$

where h_LV_ (kJ kg^−1^) is the liquid–vapor phase change enthalpy of H_2_O, and P_light_ (W m^−2^) is the solar irradiance.

**Salinity-dependent PTMD performance evaluation**
To further evaluate the desalination performance of ZLUC NFMs under seawater-relevant salinity conditions, NaCl feed solutions with different salinities were prepared by dissolving analytical-grade NaCl in deionized water. The tested NaCl concentrations were 5800, 10000, 20000, and 35000 ppm, corresponding to 5.8, 10.0, 20.0, and 35.0 g L^-1^, respectively. Among them, 5800 ppm corresponds to the originally used 0.1 M NaCl solution, while 35000 ppm was selected to approximate typical seawater-level salinity. For the salinity-dependent PTMD tests involving photocatalytic H_2_ evolution, only the NaCl concentration was varied, while the Na_2_S/Na_2_SO_3_ sacrificial-agent concentrations, membrane module, illumination condition, operating temperature, vacuum condition, effective membrane area, and other operating parameters were kept identical to those used in the standard PTMD test.

**Photoelectrochemical measurements**

The photoelectrochemical characteristics of samples in a 0.2 mol L^−1^ Na_2_SO_4_ solution were measured using a CHI 660E workstation equipped with a three-electrode system. For the irradiation light source, we employed a Xe lamp (300 W) equipped with a filter (λ > 420 nm). The Ag/AgCl, Pt foil, and samples served as reference electrodes, counter electrodes, and working electrodes, respectively. Two types of working electrodes were used. One was NFMs (1.0 cm × 2.0 cm) fixed by Nafion solution on a conducting glass to facilitate the assessment of transient photocurrent (TP) and electrochemical impedance spectroscopy (EIS). The other variant was utilized for Mott-Schottky evaluations of the catalyst powder. A slurry was generated by dispersing 5 mg of catalyst powder in 475 μL of ethanol and adding 25 μL of Nafion, following a typical protocol. Afterward, a 500 μL sample of the prepared slurry was deposited onto the surface of an ITO glass substrate measuring 1.0 cm × 2.0 cm and then allowed to air-dry. TP data were collected at 15 s intervals, while EIS examinations covered the frequency spectrum from 10^−1^ to 10^5^ Hz. Similarly, Mott-Schottky plots were produced at 500, 1000, and 1500 Hz.

**Apparent quantum yield (AQY) calculation**

The AQY for H_2_ generation was determined through controlled illumination experiments using monochromatic 400 nm excitation. This wavelength-specific measurement enables precise evaluation of the photocatalytic system's charge transfer efficiency by correlating photon absorption with product formation.

**Equation S1.** The equation of calculate AQY

AQY (%) = number of evolved (H2) molecules × 2/ number of total electrons

$$\text{ AQY = }\frac{{\text{v}\text{ }\text{× }\text{N}_{\text{A}}\text{ }\text{× }\text{K}\text{ × h × }\text{c}\text{ ×}\text{10}}^{\text{9}}}{\text{I}\text{ }\text{× }\text{A}\text{ × }\text{λ}}\text{ }$$

The reaction rate (*v*) =1.96×10^-8^ mol s^-1^

Avogadro's constant (*NA*)=6.02×10^23^ mol^-1^

The number of electrons transferred by the reaction (*K*)=2

Planck constant (*h*)= 6.626×10^-34^ J·s

Velocity of light (*c*) = 3×10^8^ m s^-1^

Light intensity (*I*) = 1000 W m^-2^

Area (*A*) =0.0045 m^2^

*λ* = 400 nm.

**Calculation of Solar-to-Hydrogen (STH) Efficiency**

The STH efficiency represents the overall conversion efficiency of solar energy into chemical energy in the form of hydrogen. It is calculated according to the following equation:

$$\text{STH = }\frac{\text{Energy output of }\text{H}_{\text{2}}}{\text{Incident solar energy}}\text{=}\frac{\text{r}_{\text{H2}}\text{ }\text{× Δ}\text{G}_{\text{H2}}}{\text{P}_{\text{light}}}\text{ }\text{× 100\% }$$

where:

*r*_H2_ is the hydrogen evolution rate (mol m^-2^ s^-1^),

Δ*G*_H2_ is the Gibbs free energy for hydrogen formation (237,000 J mol^-1^),

*P*_light_ is the incident solar power density (1000 W m^-2^, equivalent to 1 sun illumination).

#### Theoretical Calculations

All density functional theory (DFT) calculations were performed using the Vienna Ab initio Simulation Package (VASP). The exchange-correlation interactions were approximated with the Perdew-Burke-Ernzerhof (PBE) functional under the generalized gradient approximation (GGA). Electron-core interactions were modeled using the projector augmented-wave (PAW) method. A plane-wave basis set with a cutoff energy of 450 eV was applied for all computations. Structural optimizations were conducted until convergence criteria were met, with Hellmann-Feynman forces reduced below 0.03 eV/Å and energy changes minimized to less than 1×10^-4^ eV. To account for van der Waals interactions in adsorption systems, Grimme's DFT-D3 dispersion correction was incorporated. For Brillouin zone integration, a Γ-centered k-point mesh of 1×1×1 was adopted. A vacuum layer of 13 Å was introduced along the z-axis to eliminate periodic boundary effects.

**Finite-Difference Time-Domain (FDTD) Simulation**

FDTD simulations were carried out using the commercial software FDTD Solutions (Ansys Lumerical) to investigate the light–matter interaction behaviors of the ZLUC and PUC structures.

For the ZLUC model, curved fiber geometries were constructed to simulate the realistic morphology of the ZL fibrous. A coaxial shell was introduced on the surface of each fiber to represent the in situ grown MOF catalyst layer, with an average thickness of 100 nm. To better approximate the non-uniformity of the actual MOF coating, a random thickness variation of ± 25 nm was applied to the shell layer. Multiple fibers were randomly arranged and stacked to form a clustered three-dimensional fibrous network. Periodic boundary conditions were applied along the X and Y directions to simulate a large-area layered fibrous membrane, while perfectly matched layers (PML) were employed along the Z direction to eliminate artificial reflections at the simulation boundaries.

For the PUC model, a planar substrate was constructed, and a continuous MOF film layer was added on top of the substrate to mimic the configuration of powder catalysts coated on a flat PVDF substrate, enabling a direct comparison with the ZLUC structure under identical optical conditions.

A broadband plane-wave source was placed above the structures, with the incident wavelength ranging from 200 to 800 nm, covering the ultraviolet and visible regions. A reflection monitor was positioned above the light source to collect the reflected optical power, while a transmission monitor was placed beneath the structures to record the transmitted light. The wavelength-dependent absorption was calculated according to:

$$\begin{aligned} \text{A}\text{(}\text{λ}\text{)}\text{ }\text{=}\text{ }\text{1}\text{ }\text{-}\text{ }\text{R}\text{(}\text{λ}\text{)}\text{ }\text{-}\text{ }\text{T}\text{(}\text{λ}\text{)}\# \end{aligned}$$

where *R*(*λ*) and *T*(*λ*) represent the reflection and transmission spectra, respectively.

**Computational Fluid Dynamics (CFD) Simulations**

To provide deep micro/mesoscale insights into and visualize the evolution mechanisms of bubble dynamics (nucleation, growth, detachment, and escape) during the photocatalytic hydrogen evolution process on two-dimensional dense planar structures (PUC) and three-dimensional fiber network structures (ZLUC), a multiphysics transient numerical model was established based on the CFD platform ANSYS Fluent. This model couples incompressible laminar flow with the Level Set method for interface tracking.

1) Governing Equations and Interface Tracking Model

The fluid in the reaction system is assumed to be an incompressible Newtonian fluid, and the process is isothermal. The fluid dynamic behavior within the system is governed by the continuity equation and the incompressible Navier-Stokes momentum equation:

Mass conservation (continuity) equation:

$$\begin{aligned} \text{∇∙}\text{u}\text{ = 0}\# \end{aligned}$$

Momentum conservation equation:

$$\begin{aligned} \text{ }\text{ρ}\text{(}\text{ϕ}\text{) (}\frac{\text{∂}\text{u}}{\text{∂}\text{t}}\text{ }\text{+ }\text{u}\text{∙∇}\text{u}\text{) = -∇}\text{P}\text{ + ∇∙[}\text{μ}\text{(}\text{ϕ}\text{) (∇}\text{u}\text{ + }{\text{(∇}\text{u}\text{)}}^{\text{T}}\text{)] + }\text{ρ}\text{(}\text{ϕ}\text{)}\text{g}\text{ +}{\text{ }\text{F}}_{\text{sf}}\# \end{aligned}$$

where $\text{u}$ is the fluid velocity vector, *t* is time, *P* is pressure, *g* is the gravitational acceleration, and *F_sf_* is the volumetric force source term at the gas-liquid interface induced by surface tension.

To capture the dynamic evolution of the gas-liquid two-phase interface with high fidelity, the Level Set method is employed for interface tracking. By introducing a smooth phase-field indicator function $\text{ϕ}$(where the gas phase is defined as $\text{ϕ}$ = 0, and the liquid water domain is defined as $\text{ϕ}$ = 1), the convective transport of the interface is governed by the following equation:

$$\begin{aligned} \frac{\text{∂}\text{ϕ}}{\text{∂}\text{t}}\text{ }\text{+ }\text{u}\text{∙∇}\text{ϕ}\text{ }\text{= 0}\# \end{aligned}$$

The fluid properties (density $\text{ρ}$ and dynamic viscosity $\text{μ}$) at the two-phase interface undergo a smooth transition based on the indicator function $\text{ϕ}$ to ensure computational convergence:

$$\begin{aligned} \text{ρ}\text{(}\text{ϕ}\text{) =}{\text{ }\text{ρ}}_{\text{g}}\text{+ (}\text{ρ}_{\text{l}}\text{ - }\text{ρ}_{\text{g}}\text{)}\text{ϕ}\# \end{aligned}$$

$$\begin{aligned} \text{μ}\text{(}\text{ϕ}\text{) = }\text{μ}_{\text{g}}\text{+(}\text{μ}_{\text{l}}\text{ }\text{-}{\text{ }\text{μ}}_{\text{g}}\text{)}\text{ϕ}\# \end{aligned}$$

where the subscripts l and g represent the liquid phase (water) and gas phase (hydrogen), respectively.

2) Surface Tension Model

The surface tension at the gas-liquid interface is treated using the Continuum Surface Force model proposed by Brackbill, which converts it into the volumetric force source term *F_sf_* in the momentum equation:

$$\begin{aligned} \text{F}_{\text{sf}}\text{ }\text{= }\text{σ}\text{κδ}\text{(}\text{ϕ}\text{)∇}\text{ϕ}\# \end{aligned}$$

where $\text{σ}$ is the actual surface tension coefficient between the gas and liquid phases, $\text{δ}\text{(}\text{ϕ}\text{)}$ is the Dirac delta function, and $\text{κ}$ is the local curvature of the interface, defined as the divergence of the interface normal vector:

$$\begin{aligned} \text{κ}\text{ }\text{= ∇∙(}\frac{\text{∇}\text{ϕ}}{\text{|}\text{∇}\text{ϕ}\text{|}}\text{)}\# \end{aligned}$$

3) Geometric Model Construction and Boundary Conditions

Corresponding computational domain models were constructed for the gas generation behaviors of the two different catalyst structures:

PUC planar bubble behavior model: A vertical channel with an upper rectangular water domain was established, and a micro gas inlet was set at the bottom center to simulate the localized generation and accumulation of hydrogen on the planar film.

ZLUC fiber surface bubble behavior model: The cross-sectional features of a single electrospun fiber were extracted. A semi-cylindrical structure with bottom chamfers (a combination of a rectangle minus a semi-circle and a sharp bottom) was adopted as the solid-phase boundary. The top of the arc was set as the gas inlet (simulating the gas-generating active site), and the upper large-space region was defined as the continuous water phase domain.

In the solver settings, the multiphysics two-phase flow properties were assigned the standard physical parameters of hydrogen (Fluid 1) and liquid water (Fluid 2). The flow field was solved using a pressure-based transient solver, and the pressure-velocity coupling was processed via the Pressure Implicit with Splitting of Operators algorithm. This ensures the precise capture of the large interfacial deformations at the exact moment of bubble detachment under extremely small-time steps.

**Figure S1.** TGA curve of the 0.5ZLP NFMs.

Within the temperature range of 30 °C to 800 °C, the 0.5ZLP NFMs display two distinct weight loss stages, leading to a total weight reduction of 40.8%. The initial weight loss of 37.8%, occurring below 500 °C, is mainly attributed to the volatilization of acetic acid as well as the decomposition and carbonization of polyvinylpyrrolidone (PVP). Within the temperature range of 500 °C to 800 °C, an additional 3% weight loss is observed, which can be attributed to the release of water molecules resulting from the dehydroxylation of NFMs and the oxidation of residual carbon. In the differential thermal analysis (DTA) curve, the broad peak detected around 200 °C corresponds to the removal of both adsorbed and bound water present in the precursor NFMs. The exothermic peaks at 258.4 °C and 301.9 °C correspond to the decomposition and carbonization of acetic acid and PVP, respectively. The endothermic peak at 366.8 °C indicates the pyrolysis of zirconium acetate, whereas the peak at 695.6 °C signifies the grain growth of ZrO_2_.

**Figure S2.** The FTIR patterns of 0.5ZLP and 0.5ZL NFMs.

Following calcination, the peaks at 1014, 1335, 1449, 1560, and 1661 cm^-1^ in the 0.5ZLP NFMs disappeared, indicating complete pyrolysis of PVP.

**Figure S3.** Surface and cross-sectional morphologies of ZrO_2_ NFMs and ZL NFMs with varying La doping levels after calcination at different temperatures.

**Figure S4.** Fiber morphology of 0.5ZLP NFMs without calcination (left) and after calcination at 800 °C (right).

**Figure S5.** Cross-sectional image of 0.5ZLP fiber.

**Figure S6.** The XRD pattern of 0.5ZLP NFMs.

**Note S1 | Phase evolution of undoped ZrO_2_ NFMs during calcination**

According to previous reports, nanoscale t-ZrO_2_ often appears as a metastable phase during the initial crystallization of amorphous zirconia due to its favorable surface energy [1, 2]. However, as the calcination temperature increases and the grain size gradually grows—particularly when the grain diameter exceeds 30 nm—the surface energy advantage of t-ZrO_2_ diminishes. Consequently, its structure becomes thermodynamically unstable, leading to a phase transition toward the monoclinic phase, which ultimately becomes the dominant crystalline phase. The average crystallite size of ZrO_2_ NFMs calcined at 800 °C, determined by the Scherrer equation analysis, was measured to be 18.2 nm - significantly below the critical size (≈30 nm) required for the t to m phase transformation. This observation demonstrates that while t-ZrO_2_ can remain metastable at intermediate temperatures when grain sizes are sufficiently small, partial t→m phase transition still inevitably occurs during cooling due to borderline-critical crystallite dimensions combined with defect-induced and stress-mediated nucleation. Consequently, the final product exhibited m-phase dominance, illustrating the delicate interplay between size effects and phase transformation kinetics. At 1000 °C, the crystallite size increased to ~36.1 nm, resulting in complete transformation to m-ZrO_2_ due to the instability of the t-phase at this scale.

**Figure S7.** The XRD pattern of 0.5ZL NFMs at 800 °C and 1000 °C.

**Note S2 | Structural evolution and lattice distortion in 0.5ZL NFMs**

The 0.5ZL NFMs exhibited a tetragonal structure following calcination at 800 °C, with peaks at 2*θ* = 30.0°, 34.5°, 35.2°, 50.0°, 59.1°, 59.8°, and 62.5°, corresponding to the (101), (002), (110), (112), (103), (211), and (202) planes of t-ZrO_2_, as identified by JCPDS CAS No. 80-0965. The average grain size decreased to 15.7 nm. Notably, no signal peaks corresponding to La_2_O_3_ were observed. A slight shift of the peak positions toward lower angles was also detected, indicating that the unit cell volume of t-ZrO_2_ increased with La doping. After calculation, it was found that the lattice constants expanded from *a* = 3.592 Å, *c* = 5.179 Å to *a* = 3.615 Å, *c* = 5.202 Å, with the corresponding unit cell volume increasing from 66.83 Å^3^ to 67.98 Å^3^. The increase in unit cell volume is attributed to the substitution of Zr^4+^ ions (radius: 0.072 nm) by La^3+^ ions (radius: 0.106 nm). At 1000 °C, sharper peaks were observed, and the average crystallite size increased slightly to ~16.8 nm.

Furthermore, charge compensation through La^3+^ doping introduces oxygen vacancies, with this defect configuration providing additional stabilization of the tetragonal structure [3]. Notably, La doping induces a pinning effect that effectively suppresses grain growth, maintaining the crystallites at the nanoscale. The synergistic interplay between these size effects and lattice stabilization mechanisms enables exceptional long-term retention of the metastable tetragonal phase at room temperature [4].

**Figure S8.** The stress-strain curve of pure ZrO_2_ NFMs.

**Figure S9.** (a) Optical image of 0.5ZL NFMs and (b) ZrO_2_ NFMs.

The 0.5ZL NFMs exhibit flexibility, allowing them to bend freely and return to their original shape, while ZrO_2_ NFMs are very brittle and lack flexibility.

**Figure S10.** (a, e) The SEM and TEM images of original UiO-66-NH_2_. The original UiO-66-NH_2_ crystals displayed a distinct octahedral shape, with dimensions varying between 100 and 150 nm. (b, f) The SEM and TEM images of CdS. CdS particles exhibited a strong tendency to aggregate. (c, g) The SEM and TEM images of unsuperhydrophobic modified UC. After the growth of CdS on the surface of UiO-66-NH_2_, no significant changes were observed in the SEM images, whereas TEM imaging revealed well-dispersed CdS nanoparticles on the UiO-66-NH_2_ surface. This uniform dispersion of CdS highlighted the beneficial role of MOF in improving the distribution of CdS nanoparticles. (d, h) The SEM and TEM images of UC. The superhydrophobically modified UC retained the octahedral morphology of UiO-66-NH_2_ parent framework, indicating that the morphological integrity was preserved during the surface modification process.

**Figure S11.** (a) SEM and (b) TEM images of original ZLU fibers.

**Figure S12.** SEM images of ZLU NFMs grown with a seed layer (a, b) and without a seed layer (c, d) at different magnifications.

**Figure S13.** EDS elemental mapping images of ZLUC NFMs for Zr, La, S, and Cd.

**Figure S14.** The FTIR patterns of CdS, UiO-66-NH_2_ and UC.

The FTIR spectrum of UC exhibited symmetric and asymmetric stretching vibrations of alkyl –CH_2_– at 2921 and 2854 cm^-1^, which were absent in the IR spectrum of pristine UiO-66-NH_2_.

**Figure S15.** The XRD patterns of UiO-66-NH_2_, UC and CdS.

The XRD patterns of pure UiO-66-NH_2_, CdS, and UC composites are shown in the **Figure S15.** The diffraction peaks of pure CdS correspond to the cubic phase CdS (JCPDS No. 10-0454). The peaks at 2*θ* values of 26.5°, 43.9°, and 52.1° correspond to the (111), (220), and (311) crystal planes, respectively [5]. The UC composite exhibits distinct diffraction peaks corresponding to both UiO-66-NH_2_ and CdS, reflecting the two-phase composition of UiO-66-NH_2_ and CdS in the composite material, further demonstrating that UiO-66-NH_2_ retains its structural integrity after coupling with CdS. Notably, the superhydrophobic modification causes no observable reduction in crystallinity or emergence of additional diffraction peaks, demonstrating the structural robustness of the UiO-66-NH_2_ framework throughout the functionalization process.

**Figure S16.** High resolution XPS spectra of N 1s.

**Figure S17.** Water droplet (0.5 mL) low sliding angle on ZLUC NFMs, captured by a high-speed camera.

**Figure S18.** The effect of abrasion length on the CA. The inset shows the variation in CA with abrasion length for ZLUC NFMs without seed-layer growth. The error bars indicate the standard deviation derived from triplicate experimental measurements.

**Figure S19.** UV-vis reflectance spectra of the NFMs.

**Figure S20.** Infrared thermography of the surface temperature of unirradiated NFMs.

**Figure S21.** Short-term algal-fouling evaluation of ZLU and ZLUC NFMs. (a) Water flux and (b) permeate conductivity of ZLU and ZLUC NFMs during 6 h PTMD operation using an algae-containing NaCl feed solution.

**Figure S22.** 2D-OCT images of (a) ZLU and (b) ZLUC NFM surfaces after algal-fouling operation, showing the hydrated algal deposition layer.

**Figure S23.** (a) Water flux and (b) permeate conductivity of ZLU and ZLUC NFMs tested without solar illumination at a feed temperature of 60 °C. All other operating parameters were kept identical to those used in the illuminated PTMD tests.

**Figure S24.** XRD patterns of ZLUC NFMs before and after long-term operation.

**Figure S25.** The SEM image of ZLUC fiber after long-term operation.

**Figure S26.** The TEM image of ZLUC fiber after long-term operation.

**Figure S27.** Water contact angle diagram of ZLUC NFMs after long-term operation.

**Calculation of Adsorption Free Energy**

The hydrogen adsorption energy (ΔE_H_) on a catalyst surface is calculated using the following expression:

$$\text{ Δ}\text{E}_{\text{H}}\text{ = }\text{E}_{\text{(}\text{surface + H)}}\text{- }\text{E}_{\left( \text{surface} \right)}\text{- }\text{E}_{\left( \text{H}_{\text{2}} \right)}\text{/2 }$$

Here, *E*_(surface + H)_ represents the total energy of the catalyst surface with an adsorbed hydrogen atom, *E*_(surface)_ is the energy of the bare catalyst surface, and *E_(H2)_* denotes the energy of a hydrogen molecule in its gaseous state.

To determine the Gibbs free energy change (Δ*G*_H*_) for hydrogen adsorption, the following formula is applied:

$$\text{ Δ}\text{G}_{\text{H*}}\text{= ∆}\text{E}_{\text{H}}\text{+∆}\text{E}_{\text{ZPE}}\text{-}\text{T}\text{∆}\text{S}_{\text{H}}\text{ }$$

In this equation:

Δ*E*_ZPE_ accounts for the zero-point energy correction between adsorbed hydrogen and gaseous hydrogen. Δ*S*_H_ represents the entropy change during adsorption.

**Figure S28.** Molecular models of UC heterojunction for DFT calculations.

**Note: Modification of Hydrophobic Chain Length**

Due to computational limitations in handling the large system size of UiO-66-NH_2_/CdS functionalized with octadecanoyl chloride (C_18_H_35_COCl), we simplified the model by replacing the long alkyl chain with hexanoyl chloride (C_6_H_11_COCl) for DFT simulations. This substitution retains the essential chemical functionality (acyl chloride grafting) while significantly reducing computational cost. Although the chain length differs, this approximation allows us to reasonably assess the electronic structure and interfacial interactions within the heterojunction system.

**Figure S29.** Mott-Schottky plots of (a) UiO-66-NH_2_ and (c) CdS powder at different frequencies of 500, 1000, and 1500 Hz; Tauc plots and band gaps of (b) UiO-66-NH_2_ and (d) CdS powder.

**Table S1**. Key MD-relevant membrane parameters of ZL, ZLU and ZLUC NFMs.

| Substrate material | ZL | ZLU | ZLUC |
| --- | --- | --- | --- |
| Thickness (μm) | 94 | 102 | 104 |
| Porosity (%) | 88.8 | 83.4 | 82.6 |
| Mean pore size (μm) | 0.73 | 0.58 | 0.56 |
| LEP (kPa) | N.A. | 184 | 188 |
| Thermal conductivity (W m^-1^ K^-1^) | 0.071 | 0.082 | 0.086 |
| Water contact angle before (°) | 64 | 153 | 152 |
| Water contact angle after 60 h (°) | N.A. | 151.6 | 151.1 |

**Table S2**. Water-vapor fugacity driving force and ion-specific rejection of ZLUC NFMs during VMD operation.

| Parameter / ion | Feed-side value | Permeate-side value | Driving force / rejection |
| --- | --- | --- | --- |
| Water activity, *a_W_* | 0.986 | N.A. | N.A. |
| Water-vapor fugacity, *f_w_* (kPa) | 19.67 | 2.34 | $\text{Δ}\text{f}_{\text{w}}\text{ }\text{=}$17.33 kPa |
| Water flux, J ( L m^-2^ h^-1^) | N.A. | N.A. | 41.0 |
| Vapor-transport coefficient,  J/$\text{Δ}\text{f}_{\text{w}}$(L m^-2^ h^-1^ kPa^-1^) | N.A. | N.A. | 2.37 |
| Na^+^ (mg L^-1^) | 11495 | 0.68 | 99.99% |
| Cl^-^ (mg L^-1^) | 3545 | 0.21 | 99.99% |
| Total S (mg L^-1^) | 6413 | 0.37 | 99.99% |

Note:The feed-side ion concentrations were calculated based on 0.1 M NaCl, 0.1 M Na_2_S, and 0.1 M Na_2_SO_3_. Total S represents sulfur-containing species derived from Na_2_S/Na_2_SO_3_. Because VMD is governed by liquid–vapor phase change, the reported ion-specific values represent individual ion rejection rather than charge- or size-based ion selectivity. The fugacity coefficient of water vapor was approximated as unity under the low-pressure VMD conditions.

**Table S3**. PTMD desalination and photocatalytic H_2_ evolution performance of ZLUC NFMs under different NaCl salinities

| Parameter | NaCl solution | | | |
| --- | --- | --- | --- | --- |
| Salinity (ppm) | 5800 | 10000 | 20000 | 35000 |
| Flux (L m^-2^ h^-1^) | 40.8 | 37.5 | 35.1 | 32.6 |
| Permeate conductivity  (μS cm^-1^) | 4.8 | 5.7 | 7.2 | 9.1 |
| Salt rejection (%) | 99.95 | 99.96 | 99.97 | 99.98 |
| H_2_ evolution rate (mmol m^-2^ h^-1^) | 73.2 | 72.0 | 68.7 | 65.2 |
| Time (h) | 12 | 12 | 12 | 12 |

**Table S4**. Comparison of the MD performance of NFMs

| Substrate material | CA (°) | MD configuration | Rejection (%) | Flux  (L m^-2^ h^-1^) | Time (h) | Ref |
| --- | --- | --- | --- | --- | --- | --- |
| PVDF | 130 | AGMD | 99 | 11 | 600 | [6] |
| PVDF | 124 | AGMD | N.A. | 26 | 30 | [7] |
| PVDF | 139 | DCMD | 99.4 | 28 | 25 | [8] |
| PVDF | 138 | DCMD | 99 | 21 | 15 | [9] |
| PVDF | 145.8 | DCMD | 99.9 | 58 | 65 | [10] |
| PVDF | 139.6 | DCMD | N.A. | 12 | 20 | [11] |
| PVDF/MAF-4 | 147.5 | DCMD | N.A. | 27 | 6 | [12] |
| PVDF-HFP/ZIF-71 | 134 | DCMD | 99.8 | 20 | 3 | [13] |
| PVDF-HFP/SiF | >90 | DCMD | 99.99 | 26 | 11 | [14] |
| PVDF/PSf | 140 | DCMD | 99.9 | 48 | N.A. | [15] |
| PVDF-HFP/N6 | 126.3 | AGMD | 99 | 15 | 20 | [16] |
| PEBAX/Si NPs | 116.5 | VMD | N.A. | 23 | 14 | [17] |
| PAN/PVDF-HFP-PS/PDMS | 148.5 | DCMD | 100 | 27 | 36 | [18] |
| PVDF | 154 | DCMD | 99.97 | 5 | 8 | [19] |
| PVDF/PVDF/PET | 145 | AGMD | 99.9 | 15 | 40 | [20] |
| PVDF@Ag | 153 | DCMD | 99.99 | 31.6 | 8 | [21] |
| PVDF@Si NPs | 156 | DCMD | N.A. | 18.1 | 50 | [22] |
| PVDF@Si NPs | 154 | DCMD | 99 | 24.6 | 25 | [23] |
| PVDF@Si NPs | 154 | VMD | 99.9 | 11.5 | 22 | [24] |
| Substrate material | CA (°) | MD configuration | Rejection (%) | Flux  (L m^-2^ h^-1^) | Time (h) | Ref |
| PVDF@Si NPs | 155 | DCMD | 99.99 | 20.8 | 24 | [25] |
| PVDF@SiO_2_ NPs | 170 | DCMD | 99.99 | 19.1 | 8 | [26] |
| PVDF@PDMS-TiO_2_ | 150 | DCMD | 99.99 | 34.5 | 24 | [27] |
| PVDF-HFP@CNTs | 141 | DCMD | 99.9 | 18.5 | 4 | [28] |
| PDMS/ PVDF@Si NPs | 170 | DCMD | 99.99 | 28 | 160 | [29] |
| PVDF-HFP@GO NPs | 162 | AGMD | 99.9 | 22.91 | 60 | [30] |
| PVDF-HFP@TiO_2_ NPs | 149 | DCMD | N.A. | 37.8 | 48 | [31] |
| PVDF-HFP@TiO_2_ NPs | >150 | DCMD | N.A. | 38.7 | 24 | [32] |
| PVA@FAS | 158 | VMD | N.A. | 25.2 | N.A. | [33] |
| ZrO_2_@UiO-66-NH_2_/CdS | 152 | VMD | 99.95 | 41.0 | 60 | This work |

**Notes：**

Abbreviation List

PVDF: Poly (vinylidene fluoride)

SiF: [Silica](https://www.sciencedirect.com/topics/materials-science/silicon-dioxide) fibers

PSf:[Polysulfone](https://www.sciencedirect.com/topics/chemical-engineering/polysulfone)

PEBAX: Polyether block amide

GO: [Graphene oxide](https://www.sciencedirect.com/topics/materials-science/graphene-oxide)

HFP: Hexafluoropropylene

CNTs:Carbon nanotube

FAS: Perfluorooctyltriethoxysilane

NPs: [Nanoparticles](https://www.sciencedirect.com/topics/chemical-engineering/nanoparticle)

Except for the feed temperature of 70 °C in reference [26], the feed temperature of all studies is 60 °C.

References [6]–[17] are unmodified NFMs, while [18]–[33] are superhydrophobically modified NFMs. Here, the material before the "@" symbol represents the substrate, and the part after "@" denotes the modifying additive.

**References**

[1]	Rodaev VV, Zhigachev AO, Golovin YI, “Microstructure and Phase Composition of CaO-Doped Zirconia Nanofibers,” Ceramics International 43, no. 1 (2017): 1200-1204. <https://doi.org/10.1016/j.ceramint.2016.10.063>

[2]	Samson K, Sliwa M, Socha RP, et al., “Influence of ZrO_2_ Structure and Copper Electronic State on Activity of Cu/ZrO_2_ Catalysts in Methanol Synthesis from CO_2_,” Acs Catalysis 4, no. 10 (2014): 3730-3741. <https://doi.org/10.1021/cs500979c>

[3]	Dong YH, Huang YM, Ding D, Wu W, Yao X, Li J, “Chemical and Structural Origin of Hole States in Yttria-Stabilized Zirconia,” Acta Materialia 203, no. (2021): 116487. <https://doi.org/10.1016/j.actamat.2020.116487>

[4]	Claussen N, “Strengthening Strategies for ZrO_2_-Toughened Ceramics at High Temperatures,” Materials Science and Engineering 71, no. 1-2 (1985): 23-38. <https://doi.org/10.1016/0025-5416(85)90203-4>

[5]	Zhang N, Yang M-Q, Tang Z-R, Xu Y-J, “Toward Improving the Graphene-Semiconductor Composite Photoactivity via the Addition of Metal Ions as Generic Interfacial Mediator,” Acs Nano 8, no. 1 (2014): 623-633. <https://doi.org/10.1021/nn405242t>

[6]	Feng C, Khulbe KC, Matsuura I, et al., “Production of Drinking Water from Saline Water by Air-Gap Membrane Distillation Using Polyvinylidene Fluoride Nanofiber Membrane,” Journal of Membrane Science 311, no. 1-2 (2008): 1-6. <https://doi.org/10.1016/j.memsci.2007.12.026>

[7]	Moradi R, Karimi-Sabet J, Shariaty-Niassara M, Amini Y, “Air Gap Membrane Distillation for Enrichment of H₂¹⁸O Isotopomers in Natural Water Using Poly(vinylidene fluoride) Nanofibrous Membrane,” Chemical Engineering and Processing 100, no. (2016): 26-36. <https://doi.org/10.1016/j.cep.2015.11.015>

[8]	Essalhi M, Khayet M, “Self-Sustained Webs of Polyvinylidene Fluoride Electrospun Nanofibers at Different Electrospinning Times: 1. Desalination by Direct Contact Membrane Distillation,” Journal of Membrane Science 433, no. (2013): 167-179. <https://doi.org/10.1016/j.memsci.2013.01.023>

[9]	Liao Y, Wang R, Tian M, Qiu C, Fane AG, “Fabrication of Polyvinylidene Fluoride (PVDF) Nanofiber Membranes by Electrospinning for Direct Contact Membrane Distillation,” Journal of Membrane Science 425, no. (2013): 30-39. <https://doi.org/10.1016/j.memsci.2012.09.023>

[10]	Essalhi M, Khayet M, Ismail N, Sundman O, Tavajohi N, “Improvement of Nanostructured Electrospun Membranes for Desalination by Membrane Distillation Technology,” Desalination 510, no. (2021): 115086. https://doi.org/10.1016/j.desal.2021.115086

[11]	Hu X, Chen X, Giagnorio M, et al., “Beaded Electrospun Polyvinylidene Fluoride (PVDF) Membranes for Membrane Distillation (MD),” Journal of Membrane Science 661, no. (2022): 120850. <https://doi.org/10.1016/j.memsci.2022.120850>

[12]	Wu R, Tan Y, Meng F, Zhang Y, Huang Y-X, “PVDF/MAF-4 Composite Membrane for High Flux and Scaling-Resistant Membrane Distillation,” Desalination 540, no. (2022): 116013. https://doi.org/10.1016/j.desal.2022.116013

[13]	Huang M, Song J, Deng Q, Mu T, Li J, “Novel Electrospun ZIF/PcH Nanofibrous Membranes for Enhanced Performance of Membrane Distillation for Salty and Dyeing Wastewater Treatment,” Desalination 527, no. (2022): 115563. https://doi.org/10.1016/j.desal.2022.115563

[14]	Makanjuola O, Anis SF, Hashaikeh R, “Enhancing DCMD Vapor Flux of PVDF-HFP Membrane with Hydrophilic Silica Fibers,” Separation and Purification Technology 263, no. (2021): 118361. https://doi.org/10.1016/j.seppur.2021.118361

[15]	Khayet M, Garcia-Payo MC, Garcia-Fernandez L, Contreras-Martinez J, “Dual-Layered Electrospun Nanofibrous Membranes for Membrane Distillation,” Desalination 426, no. (2018): 174-184. <https://doi.org/10.1016/j.desal.2017.10.036>

[16]	Woo YC, Tijing LD, Park MJ, et al., “Electrospun Dual-Layer Nonwoven Membrane for Desalination by Air Gap Membrane Distillation,” Desalination 403, no. (2017): 187-198. <https://doi.org/10.1016/j.desal.2015.09.009>

[17]	Rasekh A, Raisi A, “Electrospun Nanofibrous Polyether-Block-Amide Membrane Containing Silica Nanoparticles for Water Desalination by Vacuum Membrane Distillation,” Separation and Purification Technology 275, no. (2021): 119149. https://doi.org/10.1016/j.seppur.2021.119149

[18]	Li S, Li L, Zhong J, et al., “Engineering Beads-on-String Structural Electrospun Nanofiber Janus Membrane with Multi-Level Roughness for Membrane Distillation,” Desalination 539, no. (2022): 115950. https://doi.org/10.1016/j.desal.2022.115950

[19]	Prince JA, Singh G, Rana D, Matsuura T, Anbharasi V, Shanmugasundaram TS, “Preparation and Characterization of Highly Hydrophobic Poly(vinylidene fluoride)-Clay Nanocomposite Nanofiber Membranes (PVDF-Clay NNMs) for Desalination Using Direct Contact Membrane Distillation,” Journal of Membrane Science 397, no. (2012): 80-86. <https://doi.org/10.1016/j.memsci.2012.01.012>

[20]	Prince JA, Anbharasi V, Shanmugasundaram TS, Singh G, “Preparation and Characterization of Novel Triple Layer Hydrophilic-Hydrophobic Composite Membrane for Desalination Using Air Gap Membrane Distillation,” Separation and Purification Technology 118, no. (2013): 598-603. <https://doi.org/10.1016/j.seppur.2013.08.006>

[21]	Liao Y, Wang R, Fane AG, “Engineering Superhydrophobic Surface on Poly(vinylidene fluoride) Nanofiber Membranes for Direct Contact Membrane Distillation,” Journal of Membrane Science 440, no. (2013): 77-87. <https://doi.org/10.1016/j.memsci.2013.04.006>

[22]	Liao Y, Wang R, Fane AG, “Fabrication of Bioinspired Composite Nanofiber Membranes with Robust Superhydrophobicity for Direct Contact Membrane Distillation,” Environmental Science and Technology 48, no. 11 (2014): 6335-6341. <https://doi.org/10.1021/es405795s>

[23]	Liao Y, Loh C-H, Wang R, Fane AG, “Electrospun Superhydrophobic Membranes with Unique Structures for Membrane Distillation,” Acs Applied Materials & Interfaces 6, no. 18 (2014): 16035-16048. <https://doi.org/10.1021/am503968n>

[24]	Yadav P, Farnood R, Kumar V, “Superhydrophobic Modification of Electrospun Nanofibrous Si@PVDF Membranes for Desalination Application in Vacuum Membrane Distillation,” Chemosphere 287, no. (2022): 132092. https://doi.org/10.1016/j.chemosphere.2021.132092

[25]	Li X, Yu X, Cheng C, Deng L, Wang M, Wang X, “Electrospun Superhydrophobic Organic/Inorganic Composite Nanofibrous Membranes for Membrane Distillation,” Acs Applied Materials & Interfaces 7, no. 39 (2015): 21919-21930. <https://doi.org/10.1021/acsami.5b06509>

[26]	Lin J, Du J, Xie S, et al., “Durable Superhydrophobic Polyvinylidene Fluoride Membranes via Facile Spray-Coating for Effective Membrane Distillation,” Desalination 538, no. (2022): 115925. https://doi.org/10.1016/j.desal.2022.115925

[27]	Liu D, Qiu M, Zhang G, Cao J, Hong Y, “Enhanced Properties of PVDF Nanofibrous Membrane with Liquid-like Coating for Membrane Distillation,” Separation and Purification Technology 295, no. (2022): 121282. https://doi.org/10.1016/j.seppur.2022.121282

[28]	Lin J, Tian F, Ding B, Yu J, Wang J, Raza A, “Facile Synthesis of Robust Amphiphobic Nanofibrous Membranes,” Applied Surface Science 276, no. (2013): 750-755. <https://doi.org/10.1016/j.apsusc.2013.03.164>

[29]	Liao Y, Zheng G, Huang JJ, Tian M, Wang R, “Development of Robust and Superhydrophobic Membranes to Mitigate Membrane Scaling and Fouling in Membrane Distillation,” Journal of Membrane Science 601, no. (2020): 117962. https://doi.org/10.1016/j.memsci.2020.117962

[30]	Woo YC, Tijing LD, Shim W-G, et al., “Water Desalination Using Graphene-Enhanced Electrospun Nanofiber Membrane via Air Gap Membrane Distillation,” Journal of Membrane Science 520, no. (2016): 99-110. <https://doi.org/10.1016/j.memsci.2016.07.049>

[31]	Lee E-J, An AK, He T, Woo YC, Shon HK, “Electrospun Nanofiber Membranes Incorporating Fluorosilane-Coated TiO_2_ Nanocomposite for Direct Contact Membrane Distillation,” Journal of Membrane Science 520, no. (2016): 145-154. <https://doi.org/10.1016/j.memsci.2016.07.019>

[32]	Shahabadi SMS, Rabiee H, Seyedi SM, Mokhtare A, Brant JA, “Superhydrophobic Dual-Layer Functionalized Titanium Dioxide/Polyvinylidene Fluoride-co-Hexafluoropropylene (TiO_2_/PH) Nanofibrous Membrane for High Flux Membrane Distillation,” Journal of Membrane Science 537, no. (2017): 140-150. <https://doi.org/10.1016/j.memsci.2017.05.039>

[33]	Dong Z-Q, Wang B-J, Ma X-H, Wei Y-M, Xu Z-L, “FAS-Grafted Electrospun Poly(vinyl alcohol) Nanofiber Membranes with Robust Superhydrophobicity for Membrane Distillation,” Acs Applied Materials & Interfaces 7, no. 40 (2015): 22652-22659. <https://doi.org/10.1021/acsami.5b07454>
